# Discovery of sugarnitriles A and B, and structural reassignment of SF-2140, from a sugarcane endophytic actinomycete *Amycolatopsis* sp. JS-O27

**DOI:** 10.3762/bjoc.22.90

**Published:** 2026-07-31

**Authors:** Qiuyun Li, Ying Liu, Baoyue Wei, Yu Sun, Di Mao, Yanmin Zou, Shan Lu

**Affiliations:** 1 School of Pharmacy, Jiangsu University, 301 Xuefu Road, Zhenjiang 212013, China

**Keywords:** *Amycolatopsis* sp., indole *N*-glycosides, SF-2140, structure reassignment, sugarnitriles

## Abstract

Three rare cyano-substituted indole *N*-glycosides, SF-2140 (**1**) and its two previously undescribed derivatives, sugarnitriles A (**2**) and B (**3**), were isolated from a sugarcane endophytic actinomycete *Amycolatopsis* sp. JS-O27. The planar structures of **1**–**3** were established by HRESIMS data and detailed 1D/2D NMR spectroscopic analysis, and the conformations of the sugar moieties were deduced by the analysis of ^3^*J*_H-H_ coupling constants and NOESY data, and comparison of their electronic circular dichroism (ECD) spectra. Compounds **1** and **3** exhibited moderate to weak antibacterial activity against *Escherichia coli* at 100 μg/mL.

## Introduction

Natural products have consistently played an indispensable role in drug discovery and development, serving as a rich reservoir of lead compounds for modern pharmaceutical research [1–2]. While plants remain a primary source for the discovery of new natural products [3–7], an increasing number of novel compounds are also being continuously identified from microorganisms [8]. Among various microorganisms, plant endophytes have attracted considerable attention as they are considered to produce structurally distinct secondary metabolites from the host plants [9–10], therefore serving as a promising source for discovering new bioactive natural products.

Indole alkaloids stand out for their structural diversity and broad pharmacological properties, including anti-inflammatory [11–12], anticancer [13–14], and anti-HIV [15–16] effects. Among indole alkaloids, indole *N*-glycosides represent a unique class of indole alkaloids [17], featuring an indole aglycone connected to a sugar moiety through a nitrogen atom. A comprehensive literature search reveals that indole *N*-glycosides are relatively rare in nature. Thus far, merely twenty indole *N*‑glycosides have been isolated and structurally characterized [18–27]. SF-2140, an indole alkaloid featuring an indole *N*-glycoside skeleton with a cyano group, was first identified from *Actinomadura* sp. in 1984 [19]. In 1989, the total synthesis of SF-2140 was achieved, further confirming its structure [28]. Preliminary bioactivity studies indicated that SF-2140 exhibits weak antibacterial activity against Gram-positive and Gram-negative bacteria and antiviral activity [19]. Although the chemical structure of SF-2140 has been assigned through total synthesis and X-ray crystallography [19], the lack of ^13^C and 2D NMR data significantly hindered the discovery and structure assignment of its derivatives.

**Figure 1:**
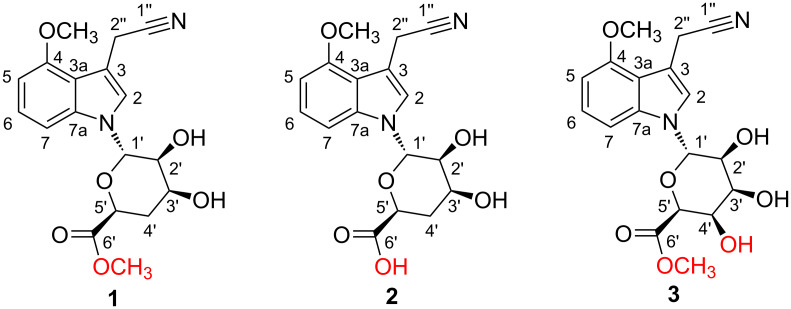
Structures of SF-2140 (**1**), sugarnitriles A (**2**) and B (**3**).

In our ongoing investigation of bioactive natural products from the endophyte library [29–33], three cyano-substituted indole *N*-glycosides, SF-2140 (**1**) and its two previously undescribed derivatives, sugarnitriles A (**2**) and B (**3**), were isolated from a sugarcane endophytic actinomycete strain *Amycolatopsis* sp. JS-O27 (Figure 1). This paper describes their isolation, structure elucidation, and biological investigations.

## Results and Discussion

HPLC analysis of the MeOH extract of rice culture of *Amycolatopsis* sp. revealed three metabolites **1**–**3** with similar UV–vis absorption. Through a large-scale fermentation (4 kg rice medium) and LCMS-guided isolation by tracing the characteristic UV absorption, compounds **1** (11.5 mg), **2** (7.5 mg), and **3** (10.0 mg) were finally obtained.

**Figure 2:**
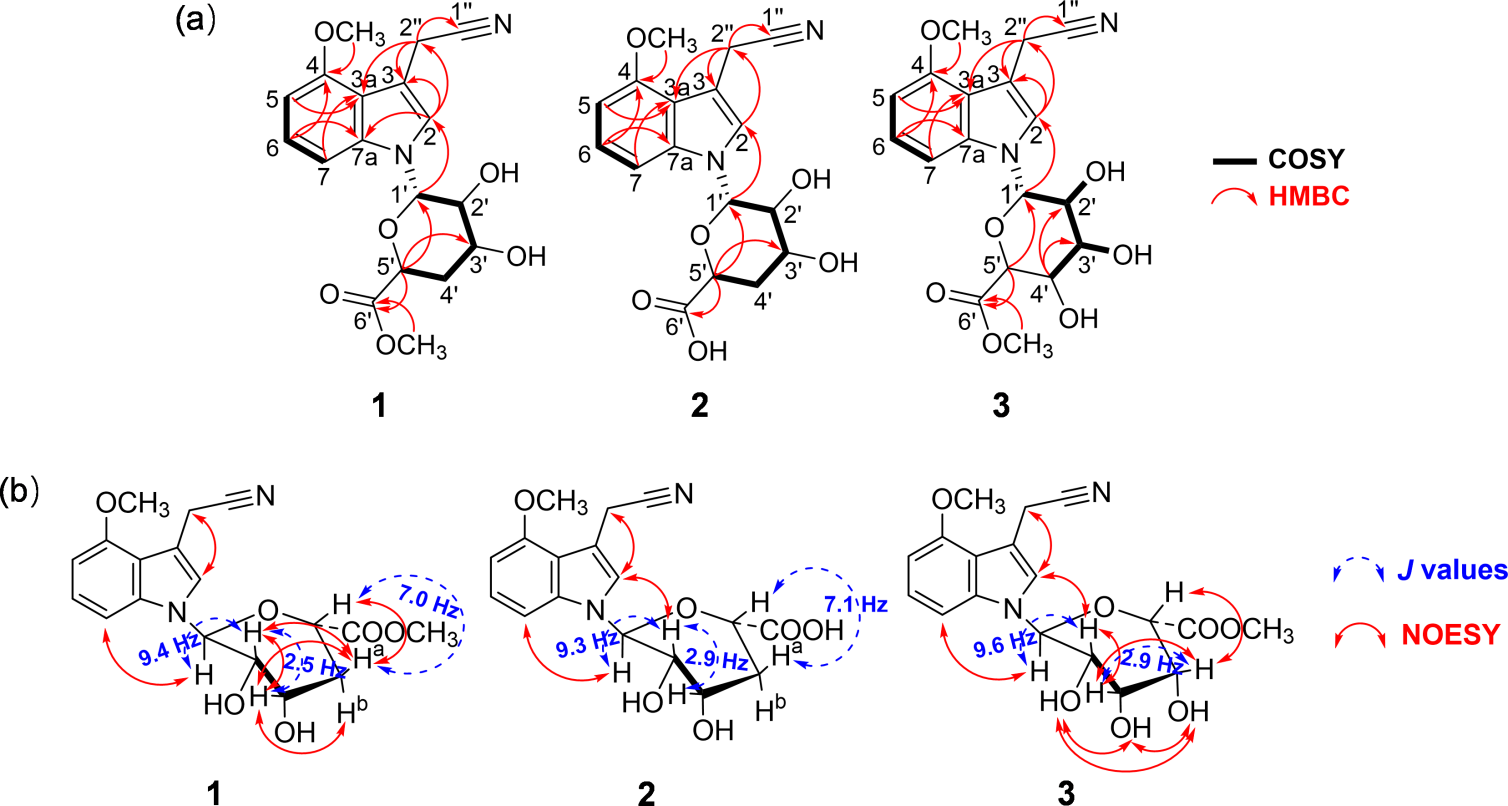
(a) ^1^H,^1^H COSY and key HMBC correlations of **1**, **2**, and **3**. (b) *J* values and key NOESY correlations of **1**, **2**, and **3**.

Compound **1** was isolated as a colorless amorphous solid. The molecular formula was determined to be C_18_H_20_N_2_O_6_ by HRESIMS (found *m*/*z* 383.1224 [M + Na]^+^, calcd. 383.1214). Analysis of the ^1^H, ^13^C, and HSQC spectra (Table 1 and Table S2, Supporting Information File 1) revealed the presence of two methoxy groups, two methylene carbons, eight methine carbons, and six quaternary carbons. The ^1^H,^1^H COSY cross peaks of H-5/H-6/H-7, together with the HMBC correlations (Figure 2a) from H-6 to C-4 and C-7a, and from H-5 and H-7 to C-3a, suggested the presence of a trisubstituted benzene ring. Meanwhile, the HMBC correlations from H-2 to C-2'', C-3, and C-7a supported the presence of a pyrrole ring, and the HMBC correlations from H-2'' to C-3a indicated that the pyrrole ring is fused with a benzene ring at C-3a and C-7a to form an indole skeleton. The DEPT135 spectrum supported the assignment of C-1'' as a quaternary carbon, and the chemical shift of C-1'' (δ_C_ 119.6 ppm) was consistent with the reported data of a cyano carbon [34]. The ^1^H,^1^H COSY signals for H-1'/H-2'/H-3'/H-4'/H-5', accompanied by the HMBC correlations from H-5' to C-1' and C-3', established a glycosyl moiety. Finally, from the HMBC signals between H-1' and C-2, it was determined that the anomeric carbon (C-1') was connected to the indole ring through a heteroatom, revealing that compound **1** is an indole *N*-glycoside (Figure 2a). Further detailed analysis of the 2D NMR data of **1** indicated that the planar structure of **1** was identical to that of SF-2140 (Tables S1 and S2, Supporting Information File 1, and Figure 2a). Since the ^13^C and 2D NMR data of SF-2140 were lacking in previous papers [19,28], the current research supplemented the missing information.

**Table 1:** ^1^H and ^13^C NMR data of **1**, **2**, and **3**.

Position	Compound **1**		Compound **2**		Compound **3**	

	δ_C_, type	δ_H_ (*J* in Hz)	δ_C_, type	δ_H_ (*J* in Hz)	δ_C_, type	δ_H_ (*J* in Hz)

2	123.0, CH	7.38, s	123.2, CH	7.35, s	123.4, CH	7.36, s
3	104.4, C	–	104.0, C	–	104.3, C	–
3a	116.4, C	–	116.3, C	–	116.5, C	–
4	153.7, C	–	153.6, C	–	153.8, C	–
4-OMe	55.3, CH_3_	3.87, s	55.3, CH_3_	3.87, s	55.4, CH_3_	3.87, s
5	100.5, CH	6.60, d, 7.8	100.3, CH	6.57, d, 7.8	100.5, CH	6.60, d, 7.8
6	123.2, CH	7.13, t, 8.1	122.9, CH	7.08, t, 8.1	123.1, CH	7.12, t, 8.1
7	104.3, CH	7.36, d, 8.5	104.8, CH	7.36, d, 8.0	104.5, CH	7.36, d, 8.4
7a	138.8, C	–	138.8, C	–	138.6, C	–
1'	78.1, CH	6.13, d, 9.4	78.4, CH	6.15, d, 9.3	78.7, CH	6.11, d, 9.6
2'	68.5, CH	3.96, dd, 9.3, 2.5	69.1, CH	3.90, dd, 9.2, 2.9	64.8, CH	4.28, d, 9.0
2'-OH	–	–	–	–	–	5.08, s
3'	67.0, CH	4.08, br s	67.3, CH	4.03, s	71.2, CH	3.94, br s
3'-OH	–	–	–	–	–	5.50, s
4'a	33.6, CH_2_	2.17, ddd, 14.0, 7.0, 1.9	33.1, CH_2_	2.07, ddd, 14.0, 7.1, 2.2	70.9, CH	4.21, d, 2.9
4'b	–	2.30, dd, 13.1, 1.9	–	2.29, dd, 13.5, 1.8	–	–
4'-OH	–	–	–	–	–	5.63, s
5'	69.3, CH	4.49, d, 6.5	71.9, CH	4.18, d, 6.9	76.0, CH	4.36, s
6'	172.4, C	–	174.7, C	–	170.2, C	–
6'-OMe	51.5, CH_3_	3.70, s	–	–	51.6, CH_3_	3.70, s
1''	119.6, C	–	119.7, C	–	119.7, C	–
2''	15.2, CH_2_	4.04, s	15.2, CH_2_	4.05, s	15.2, CH_2_	4.05, s

The sugar moiety of SF-2140 has been determined to adopt a ^1^*C*_4_ conformation in the solid state and was suggested to adopt a possible twist-boat conformation in solution based on ^3^*J*_H−H_ coupling constants [19]. The chemical shifts, ^3^*J*_H−H_ coupling constants, and NOESY data of the sugar moiety of **1** indicated that its relative configurations were the same as those of SF-2140, which adopts a twist-boat conformation in solution (Table S2, Supporting Information File 1, and Figure 2b). The large coupling constant (*J* = 9.4 Hz) between H-1' and H-2' indicated both the indole and the 2'-OH to be in pseudo-equatorial positions. Furthermore, the small coupling constants between H-2' and H-3', and between H-3' and H-4'b, and NOESY correlations of H-2'/H-3', H-2'/H-4'a, H-3'/H-4'a revealed that H-3' is oriented in the pseudo-equatorial position and is nearly orthogonal to both H-4'a and H-4'b. The negligible vicinal coupling constant between H-5' and H-4'b indicates a dihedral angle close to 90°. Therefore, the coupling constant between H-4'a and H-5' (*J* = 7.0 Hz) suggests that they are located on the same side of the ring.

The absolute configurations of SF-2140 were determined by X-ray crystallographic analysis [19] and further confirmed through a total synthesis method subsequently [28]. A comparison of the specific rotation value of **1** (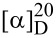 +64.3 in MeOH, *c* 0.88) with that of SF-2140 (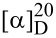 +59 in MeOH, *c* 1.0) indicated that the absolute configuration of **1** is consistent with that of SF-2140.

**Figure 3:**
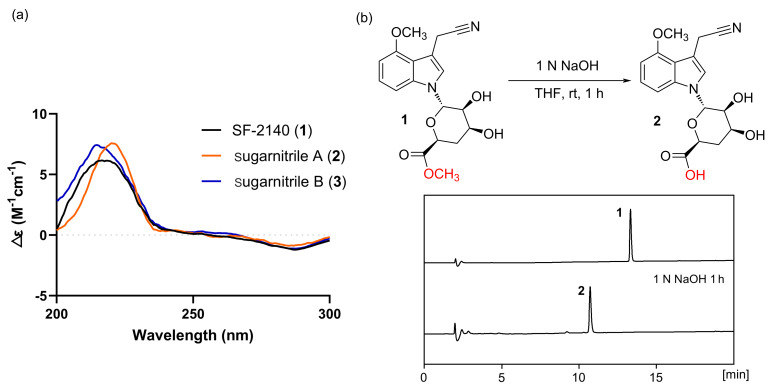
(a) Comparison of the experimental ECD spectra of **1**, **2**, and **3** in MeOH. (b) Hydrolysis of **1** to **2** (YMC Hydrosphere C18, ∅ 3 × 150 mm, 0.5 mL/min, 0–20 min, grad. 20–100% aq MeCN, PDA: 220 nm).

Compound **2** was isolated as a colorless amorphous solid (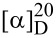 −31.0 in MeOH, *c* 0.67). The molecular formula was deduced as C_17_H_18_N_2_O_6_ by HRESIMS (found *m*/*z* 347.1238 [M + H]^+^, calcd. 347.1243). The MS signal of **2** exhibited 14 mass units less than that of **1**. A comparison of the ^1^H and ^13^C NMR data revealed that the planar structure of **2** was very similar to that of **1** except for the absence of a methyl group at C-6' in **2** (Table 1). The planar and relative structure of **2** was then elucidated by detailed analysis of 2D NMR data with a combination of analysis of ^3^*J*_H−H_ coupling constants and NOESY data (Table S3, Supporting Information File 1, and Figure 2). To determine the absolute configurations of **2**, the electronic circular dichroism (ECD) spectra of **1** and **2** were measured and compared. The curve of the ECD spectrum of **2** showed a positive Cotton effect at around 223 nm and a negative Cotton effect at around 286 nm, which matched well with those of **1** (Figure 3a), suggesting that they possess the same absolute configurations. This conclusion was further supported by the chemical conversion of **1** to **2** (Figure 3b).

Compound **3** was isolated as a colorless amorphous solid (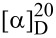 +47.4 in MeOH, *c* 1.0). Its molecular formula was determined as C_18_H_20_N_2_O_7_ by HRESIMS (found *m*/*z* 377.1352 [M + H]^+^, calcd. 377.1343). The MS data showed that compound **3** exhibited 16 mass units more than that of **1**. The ^1^H and ^13^C NMR data of **3** were identical to those of **1** in the indole ring but slightly distinct in the sugar moiety (Table 1). The CH_2_ signal (δ_C_ 33.6 in **1**, Figure S6, Supporting Information File 1) was absent in the DEPT135 spectrum, accompanied by an additional CH signal (δ_C_ 70.9, Figure S20, Supporting Information File 1), suggesting an OH group substituted at C-4'. The ^1^H,^1^H COSY cross peaks of H-3'/H-4', H-2'/2'-OH, and H-3'/3'-OH, together with HMBC correlations from H-4' to C-2' and C-3', indicated that **3** is the C-4' hydroxylated derivative of **1**. The planar structure of **3** was confirmed by detailed analysis of 2D NMR data (Table S4, Supporting Information File 1, and Figure 2).

The relative stereochemistry was deduced through ^3^*J*_H−H_ coupling constants and NOESY data analysis (Table S4, Supporting Information File 1, and Figure 2b). NOESY correlations of H-3'/H-4', H-4'/H-5', and 2'-OH/3'-OH/4'-OH suggested that H-3', H-4', and H-5' are located on the same side of the ring. Meanwhile, the small coupling constant (*J* = 2.9 Hz) between H-3' and H-4' and the negligible vicinal coupling constant between H-4' and H-5' suggested that the dihedral angles between H-3' and H-4', and between H-4' and H-5', are close to 90°. Therefore, the relative configurations of compound **3** were determined as 1'S*,2'S*,3'S*,4'R*,5'S*. The absolute configurations of **3** were then determined by comparing the ECD spectra of **3** and **1** (Figure 3a). The well-matched curves suggested they possess the same absolute configuration of C-1'. Thus, the absolute configurations of **3** were determined as 1'*S*,2'*S*,3'*S*,4'*R*,5'*S*.

**Figure 4:**
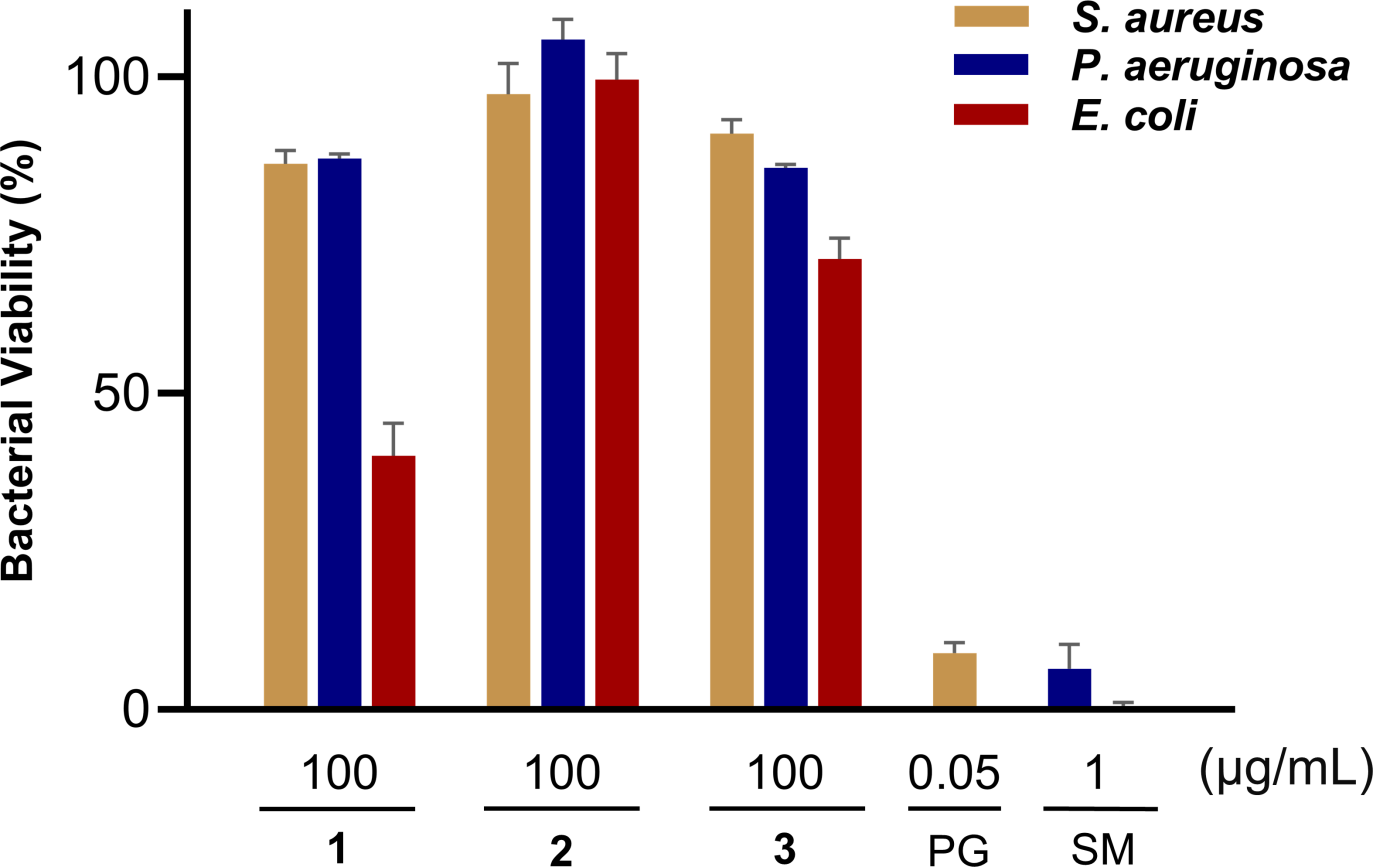
Antibacterial activity of **1**, **2**, and **3**. Positive controls: penicillin G (PG) for *S. aureus* and streptomycin (SM) for *P. aeruginosa* and *E. coli*.

We next evaluated the bioactivity of compounds **1**–**3**. In the cytotoxicity assay, the three compounds showed no cytotoxicity against HepG2 cells at 50 μM. In the antibacterial assay, Gram-positive *Staphylococcus aureus* (*S. aureus),* Gram-negative *Escherichia coli* (*E. coli*), and *Pseudomonas aeruginosa* (*P. aeruginosa*) were selected for the evaluation. Compounds **1** and **3** exhibited moderate to weak antibacterial activity against *E. coli* at 100 µg/mL, but no activity against *S. aureus*, revealing Gram-negative-bacteria-selective growth inhibition. However, further evaluation with another Gram-negative bacterium, *P. aeruginosa,* indicated that none of them were active at 100 µg/mL. Compound **2** was inactive against all the bacteria, suggesting the negative effect of the carboxyl group on the antibacterial activity (Figure 4).

## Conclusion

In conclusion, this study carried out the complete NMR assignment of SF-2140 (**1**) and identified two new derivatives, sugarnitriles A (**2**) and B (**3**), expanding the structural diversity of indole *N*-glycosides. The full NMR assignment of these compounds provides a basis for the structure elucidation of new indole *N*-glycosides in the future. Despite previous reports indicating that SF-2140 (**1**) exhibited weak to moderate activity against both Gram-positive and Gram-negative bacteria [19], compound **1** only exhibited moderate antibacterial activity against *E. coli* at 100 μg/mL in our experiments. The selective growth inhibition against *E. coli* by compounds **1** and **3** suggests their unique mode of action, highlighting the need for further study. The structural feature of indoleacetonitrile in **1**–**3** also suggests that they may work as new plant auxins. The related studies are currently underway.

## Experimental

### General experimental procedures

All reagents and solvents were purchased from commercial suppliers, including Adamas, Adamas life, and Greagent, and used as received without further drying or purification. 1D and 2D NMR spectra were recorded at 25 °C on a Bruker Avance III HD 400 NMR spectrometer. Mass and high-resolution mass spectrometry (HRMS) spectra were obtained on an Agilent 1260-6120 LC/MS spectrometer or a Waters Xevo G2-XS QT mass spectrometer. CD spectra were recorded at 25 °C on a JASCO J-815 CD spectropolarimeter with a 0.2 cm path length cell. Optical rotations were measured at 20 °C on an INESA WZZ-2B polarimeter. UV–vis spectra were acquired in MeOH on a METASH UV-8000 spectrophotometer. Analytical and preparative reversed-phase HPLC (RPLC) was conducted on a Shimadzu Prominence HPLC system using a YMC Hydrosphere C18 analytical column (3 μm, 120 Å, 150 × 4.6 mm) and a YMC Hydrosphere C18 preparative column (5 μm, 120 Å, 150 × 10 mm).

### Fermentation

A plate culture of the strain *Amycolatopsis* sp. JS-O27 was inoculated into a 250 mL Erlenmeyer flask containing 100 mL of International Streptomyces Project-2 medium (ISP2 medium, pH 7.4) for 3 days at 28 °C on a rotary shaker at 160 rpm. The seed culture (1.0 mL) was transferred into a 250 mL flask (total of 80 flasks) containing rice medium (rice medium: 50 g rice and 1 mL of trace element solution in 60 mL of water, pH 7.2) and was cultured under static conditions at 28 °C for 30 days.

### Isolation of compounds **1**, **2**, and **3**

The whole rice culture (4.0 kg) was exhaustively extracted with MeOH to afford a residue (6.2 g) after concentration in vacuo. The residue was then partitioned between water and EtOAc and exhaustively extracted to afford a crude product (2.2 g). The EtOAc extract was chromatographed on a silica gel column, eluted with a gradient mixture of *n*-hexane/EtOAc/MeOH (20:1:0 to 0:1:20), yielding 18 fractions. Fraction 7 afforded compound **1** (11.5 mg). Fraction 9 afforded compound **3** (10.0 mg). Fraction 16 afforded compound **2** (7.5 mg).

SF-2140 (**1**): colorless amorphous solid; 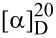 +64.3 (*c* 0.88, MeOH); CD (*c* 3.13 × 10^−4^ M, MeOH); UV(MeOH) λ_max_ (log ε) 222 (4.49), 265 (3.85), 284 (3.75), 294 (3.78) nm; 1D and 2D NMR data, see Table S2, Supporting Information File 1; HRESIMS (*m*/*z*): [M + Na]^+^ calcd for C_18_H_20_N_2_NaO_6_, 383.1214; found, 383.1224.

Sugarnitrile A (**2**): colorless amorphous solid; 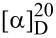 −31.0 (*c* 0.67, MeOH); CD (*c* 3.47 × 10^−4^ M, MeOH); UV(MeOH) λ_max_ (log ε) 220 (4.45), 266 (3.81), 284 (3.72), 294 (3.75) nm; 1D and 2D NMR data, see Table S3, Supporting Information File 1; HRESIMS (*m*/*z*): [M + H]^+^ calcd for C_17_H_19_N_2_O_6_, 347.1243; found, 347.1238.

Sugarnitrile B (**3**): colorless amorphous solid; 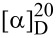 +47.4 (*c* 1.0, MeOH); CD (*c* 2.98 × 10^−4^ M, MeOH); UV(MeOH) λ_max_ (log ε) 220 (4.43), 265 (3.79), 284 (3.69), 293 (3.73) nm; 1D and 2D NMR data, see Table S4, Supporting Information File 1; HRESIMS (*m*/*z*): [M + H]^+^ calcd for C_18_H_21_N_2_O_7_, 377.1343; found, 377.1352.

### Chemical conversion of **1** to **2**

Compound **1** (1.0 mg) was hydrolyzed under conditions of 1 N aq NaOH in MeOH (1.0 mL) at rt for 1 h. A portion of the reaction mixture was sampled and analyzed by RP-HPLC (YMC Hydrosphere C18, ∅ 3 × 150 mm, 0.5 mL/min, 0–20 min, grad. 20–100% aq MeCN, PDA: 220 nm).

### Cytotoxicity

In a manner similar to our previous report [32], cytotoxicity was assayed with the CCK-8 (Dojindo, Japan) method. HepG2 cells were obtained from the School of Medicine, Jiangsu University. Cells were routinely grown and maintained in a DMEM medium with 10% FBS and 1% penicillin/streptomycin. Briefly, the cells were seeded at a density of 5000–10000 cells per well in a 96-well plate. Doxorubicin was used as a positive control. After 24 h, the cells were treated with various concentrations of compounds or the positive control. After 96 h incubation, CCK-8 reagent was added, and absorbance was measured at 450 nm.

### Antibacterial activity

In a manner similar to our previous report [32], an antibacterial assay was conducted in flat-bottom, sterile 96-well plates (Corning) in triplicate, using a broth microdilution protocol. Penicillin and streptomycin were used as positive controls for Gram-positive and Gram-negative bacteria, respectively. Compounds **1**–**3** were tested for antibacterial activity against *S. aureus*, *P. aeruginosa,* and *E. coli*. Briefly, the bacteria were cultured on an LB agar plate and incubated overnight at 28 °C. The bacteria were then transferred into 50 mL of LB medium in a 250 mL flask and cultured for 5 h at 28 °C. The cells were suspended in the same medium to produce a suspension of 10^6^ CFU/mL and then added into each well (99 μL/well) in a 96-well plate. Subsequently, 1 μL of each tested compound was added with a final concentration of 100 and 50 μg/mL. The mixture was incubated at 28 °C for 16 h, and the absorption was evaluated at 600 nm with a plate reader. The MIC values were calculated as the lowest concentration of the compound that inhibited bacterial growth.

## Supporting Information

File 1 NMR data and copies of NMR and MS spectra.

## Data Availability

All data that supports the findings of this study is available in the published article and/or the supporting information of this article.
