# Supplementary material for: Discovery of sugarnitriles A and B, and structural reassignment of SF-2140, from a sugarcane endophytic actinomycete Amycolatopsis sp. JS-O27
Source: Beilstein J Org Chem. 2026 Jul 31;22:1114–21. doi: 10.3762/bjoc.22.90 (PMC13430523; doi:10.3762/bjoc.22.90)
Supplement: File 1 — NMR data and copies of NMR and MS spectra. [file Beilstein_J_Org_Chem-22-1114-s001.pdf]

BEILSTEIN JOURNAL OF ORGANIC CHEMISTRY

Supporting Information

for

Discovery of sugarnitriles A and B, and structural reassignment of SF-2140, from a sugarcane endophytic actinomycete *Amycolatopsis* sp. JS-O27

Qiuyun Li, Ying Liu, Baoyue Wei, Yu Sun, Di Mao, Yanmin Zou and Shan Lu

Beilstein J. Org. Chem. **2026,** *22,* 1114–1121. doi:10.3762/bjoc.22.90

NMR data and copies of NMR and MS spectra

License and Terms: This is a supporting information file under the terms of the Creative Commons Attribution License (https://creativecommons.org/licenses/by/4.0). Please note that the reuse, redistribution and reproduction in particular requires that the author(s) and source are credited and that individual graphics may be subject to special legal provisions.
The license is subject to the *Beilstein Journal of Organic Chemistry* terms and conditions: (https://www.beilstein-journals.org/bjoc/terms)

Table of Contents

Table S1. Comparison of ^{1}H NMR data for reported SF-2140 and compound **1**

Table S2. 1D and 2D NMR data for **1** in DMSO-d_6

Table S3. 1D and 2D NMR data for **2** in DMSO-d_6

Table S4. 1D and 2D NMR data for **3** in DMSO-d_6

Figure S1. HRESIMS spectra of **1**

Figure S2. HRESIMS spectra of **2**

Figure S3. HRESIMS spectra of **3**

Figure S4. ^{1}H NMR spectrum (400 MHz) of **1** in DMSO-d_6

Figure S5. ^{13}C NMR spectrum (100 MHz) of **1** in DMSO-d_6

Figure S6. DEPT135 spectrum of **1** in DMSO-d_6

Figure S7. ^{1}H-^{1}H COSY spectrum of **1** in DMSO-d_6

Figure S8. HSQC spectrum of **1** in DMSO-d_6

Figure S9. HMBC spectrum of **1** in DMSO-d_6

Figure S10. NOESY spectrum of **1** in DMSO-d_6

Figure S11. ^{1}H NMR spectrum (400 MHz) of **2** in DMSO-d_6

Figure S12. ^{13}C NMR spectrum (100 MHz) of **2** in DMSO-d_6

Figure S13. DEPT135 spectrum of **2** in DMSO-d_6

Figure S14. ^{1}H-^{1}H COSY spectrum of **2** in DMSO-d_6

Figure S15. HSQC spectrum of **2** in DMSO-d_6

Figure S16. HMBC spectrum of **2** in DMSO-d_6

Figure S17. NOESY spectrum of **2** in DMSO-d_6

Figure S18. ^{1}H NMR spectrum (400 MHz) of **3** in DMSO-d_6

Figure S19. ^{13}C NMR spectrum (100 MHz) of **3** in DMSO-d_6

Figure S20. DEPT135 spectrum of **3** in DMSO-d_6

Figure S21. ^{1}H-^{1}H COSY spectrum of **3** in DMSO-d_6

Figure S22. HSQC spectrum of **3** in DMSO-d_6

Figure S23. HMBC spectrum of **3** in DMSO-d_6

Figure S24. NOESY spectrum of **3** in DMSO-d_6

Table S1: Comparison of ^{1}H NMR data for reported SF-2140 and compound **1**.

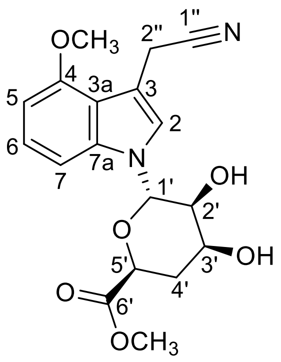

SF-2140 (**1**)

NO.	SF-2140 (acetone-d_6)		compound **1** (DMSO-d_6)
	δ_H (*J* in Hz) [a]	δ_H (*J* in Hz) [b]	δ_H (*J* in Hz)
2	lack	7.36, s	7.38, s
3	-	-	-
3a	-	-	-
4	-	-	-
4-OMe	lack	3.90, s	3.87, s
5	lack	6.57, d, 7.5	6.60, d, 7.8
6	lack	7.11, t, 8.0	7.13, t, 8.1
7	lack	7.42, d, 8.0	7.36, d, 8.5
7a	-	-	-
1'	6.33, d, 9.3	6.29, d, 9.4	6.13, d, 9.4
2'	4.16, ddd, 2.7	4.12, dd, 9.4, 2.8	3.96, dd, 9.3, 2.5
2'-OH	4.36, d, 7.1	-	-
3'	4.29, ddd, 3.7, 2.2	4.26, m	4.08, br s
3'-OH	4.21, dd, 2.7, 1.2	-	-
4'a	2.28, dddd, 14.4, 6.8	2.28, ddd, 14.3, 7.0	2.17, ddd, 14.0, 7.0, 1.9
4'b	2.51, dddd, 1.2	2.50, ddd, 1.3	2.30, dd, 13.1, 1.9
5'	4.45, br d	4.42, br d	4.49, d, 6.5
6'	-	-	-
6'-OMe	3.75, s	3.75, s	3.70, s
1"	-	-	-
2"	lack	4.02, s	4.04, s

[a] *J. Antibiot.* **1984**, *37*, 931-934.

[b] *J. Chem. Soc., Perkin Trans. 1* **1994**, 1417-1426.

Table S2: 1D and 2D NMR data for **1** in DMSO-d_6.

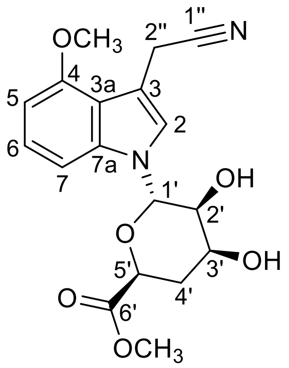

1

No.	δ_H, m, J (Hz)	δ_C, type	COSY	HMBC	NOESY
2	7.38, s	123.0, CH	2"	2", 3, 7a	2"
3	-	104.4, C			
3a	-	116.4, C			
4	-	153.7, C			
4-OMe	3.87, s	55.3, CH_3		4	5
5	6.60, d, 7.8	100.5, CH	6	3a, 4, 7	4-OMe, 6
6	7.13, t, 8.1	123.2, CH	5, 7	4, 5, 7, 7a	5, 7
7	7.36, d, 8.5	104.3, CH	6	3a, 5	1', 6
7a	-	138.8, C			
1'	6.13, d, 9.4	78.1, CH	2'	2, 2', 7a	2', 7
2'	3.96, dd, 9.3, 2.5	68.5, CH	1'	1'	1', 3', 4'a
3'	4.08, br s	67.0, CH	4'b		2', 4'a, 4'b
4'a	2.17, ddd, 14.0, 7.0, 1.9	33.6, CH_2	5'	5', 6'	2', 3', 4'b, 5'
4'b	2.30, dd, 13.1, 1.9	-	3'		3', 4'a, 5'
5'	4.49, d, 6.5	69.3, CH	4'a	1', 3', 4', 6'	4'a, 4'b
6'	-	172.4, C			
6'-OMe	3.70, s	51.5, CH_3		6'	
1"	-	119.6, C			
2"	4.04, s	15.2, CH_2	2	1", 2, 3, 3a	2

Table S3: 1D and 2D NMR data for **2** in DMSO-d_6.

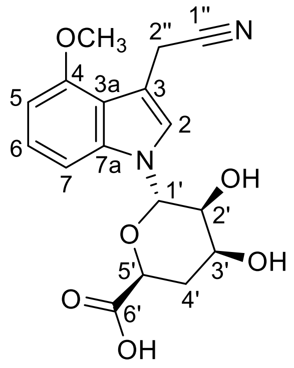

2

No.	δ_H, m, J (Hz)	δ_C, type	COSY	HMBC	NOESY
2	7.35, s	123.2, CH	2"	2"	2', 2"
3	-	104.0, C			
3a	-	116.3, C			
4	-	153.6, C			
4-OMe	3.87, s	55.3, CH_3		4	5
5	6.57, d, 7.8	100.3, CH	6	3a, 4, 7	4-OMe, 6
6	7.08, t, 8.1	122.9, CH	5, 7	4, 7a	5, 7
7	7.36, d, 8.0	104.8, CH	6	3a, 5, 7a	1', 6
7a	-	138.8, C			
1'	6.15, d, 9.3	78.4, CH	2'	2, 2', 7a	7
2'	3.90, dd, 9.2, 2.9	69.1, CH	1', 3'		2
3'	4.03, s	67.3, CH	2', 4'b		
4'a	2.07, ddd, 14.0, 7.1, 2.2	33.1, CH_2	5'		
4'b	2.29, dd, 13.5, 1.8	-	3'		
5'	4.18, d, 6.9	71.9, CH	4'a	1', 3', 6'	
6'	-	174.7, C*			
1"	-	119.7, C			
2"	4.05, s	15.2, CH_2	2	1", 2, 3, 3a	2

Note: * Assignment made by HMBC.

Table S4: 1D and 2D NMR data for **3** in DMSO-d_6.

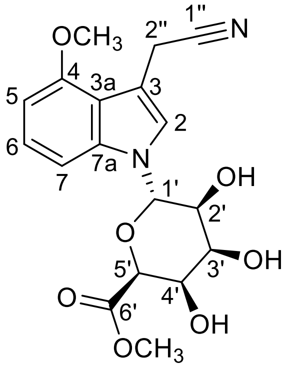

3

No.	δ_H, m, *J* (Hz)	δ_C, type	COSY	HMBC	NOESY
2	7.36, s	123.4, CH	2"	2", 1', 3	2', 2"
3	-	104.3, C			
3a	-	116.5, C			
4	-	153.8, C			
4-OMe	3.87, s	55.4, CH_3		4	5
5	6.60, d, 7.8	100.5, CH	6	3a, 4, 6	4-OMe, 6
6	7.12, t, 8.1	123.1, CH	5, 7	4, 5, 7, 7a	5, 7
7	7.36, d, 8.4	104.5, CH	6	4, 5, 3a, 7a	1', 6
7a	-	138.6, C			
1'	6.11, d, 9.6	78.7, CH	2'	2, 2', 3', 7a	2', 7
2'	4.28, d, 9.0	64.8, CH	1', 2'-OH, 3'		1', 2, 3'
2'-OH	5.08, s	-	2'		3'-OH, 4'-OH
3'	3.94, br s	71.2, CH	2', 3'-OH, 4'		2', 4'
3'-OH	5.50, s	-	3'		2'-OH, 4'-OH
4'	4.21, d, 2.9	70.9, CH	3'	2', 3'	3', 5'
4'-OH	5.63, s	-			2'-OH, 3'-OH
5'	4.36, s	76.0, CH		1', 3', 6'	4'
6'	-	170.2, C			
6'-OMe	3.70, s	51.6, CH_3		6'	
1"	-	119.7, C			
2"	4.05, s	15.2, CH_2	2	1", 2, 3, 3a,	2

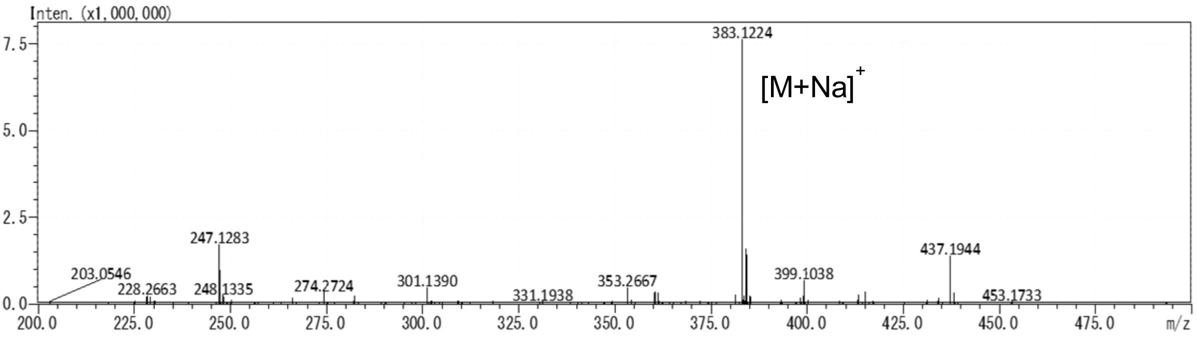

Figure S1: HRESIMS spectrum of **1**.

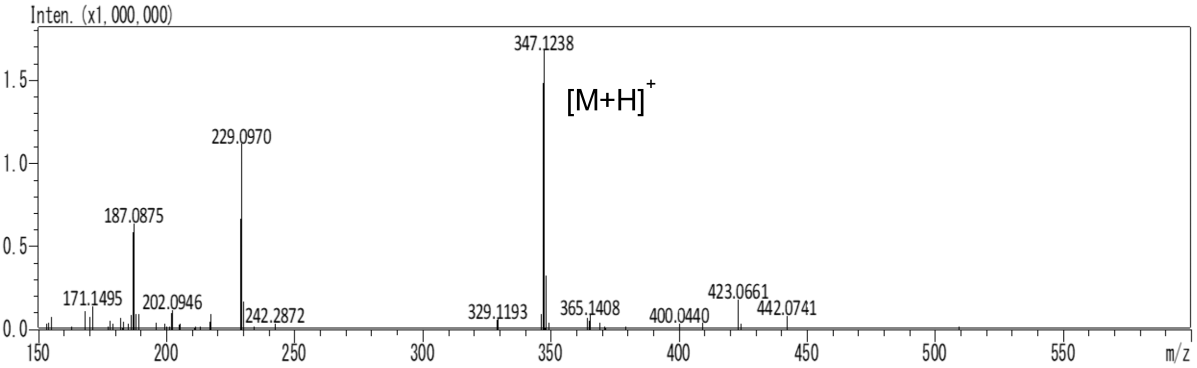

Figure S2: HRESIMS spectrum of **2**.

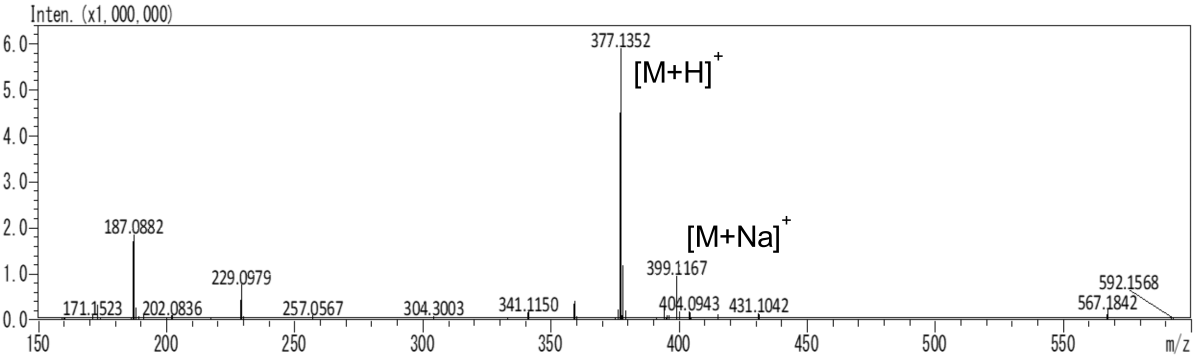

Figure S3: HRESIMS spectrum of **3**.

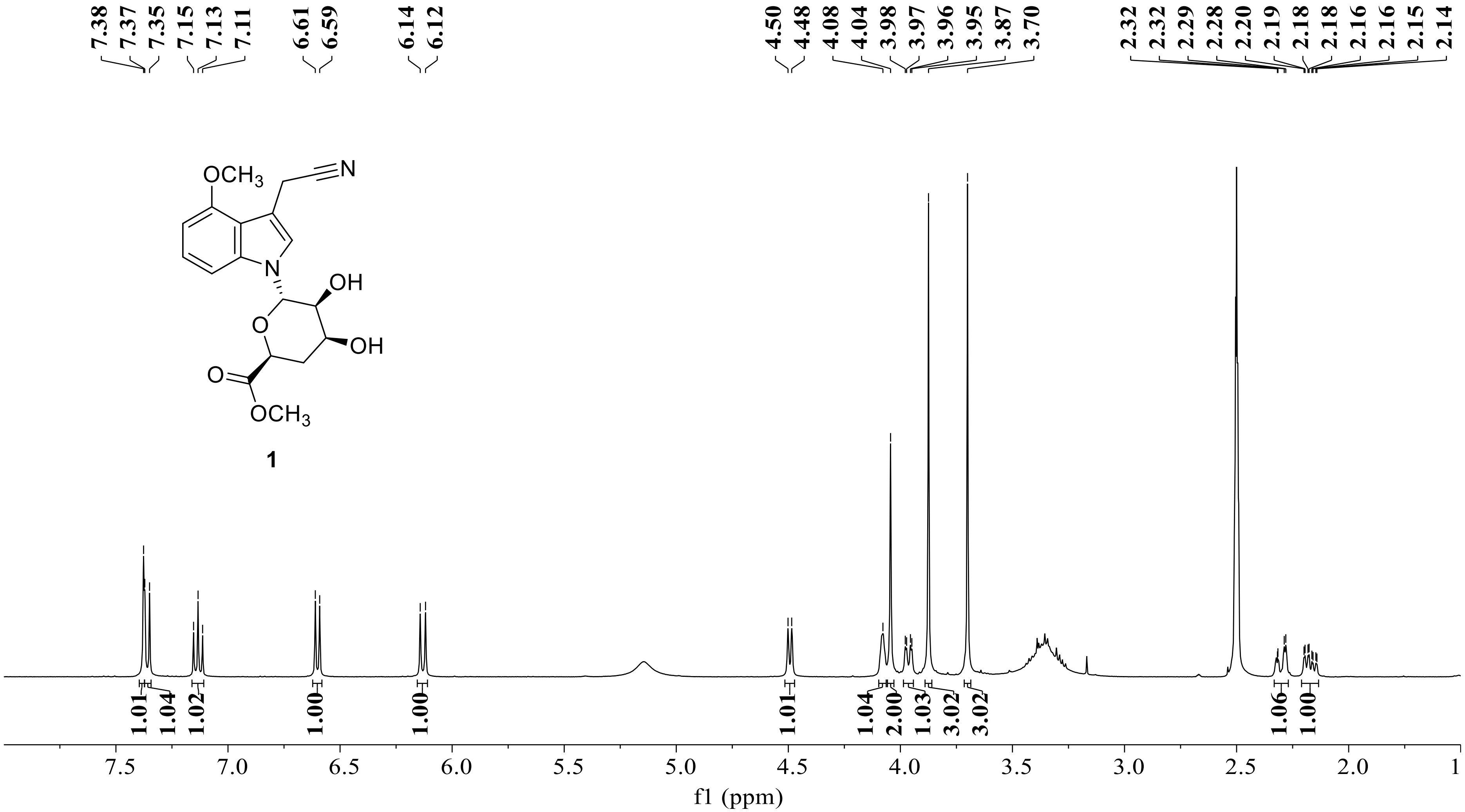

Figure S4: ^{1}H NMR spectrum (400 MHz) of **1** in DMSO-d_6.

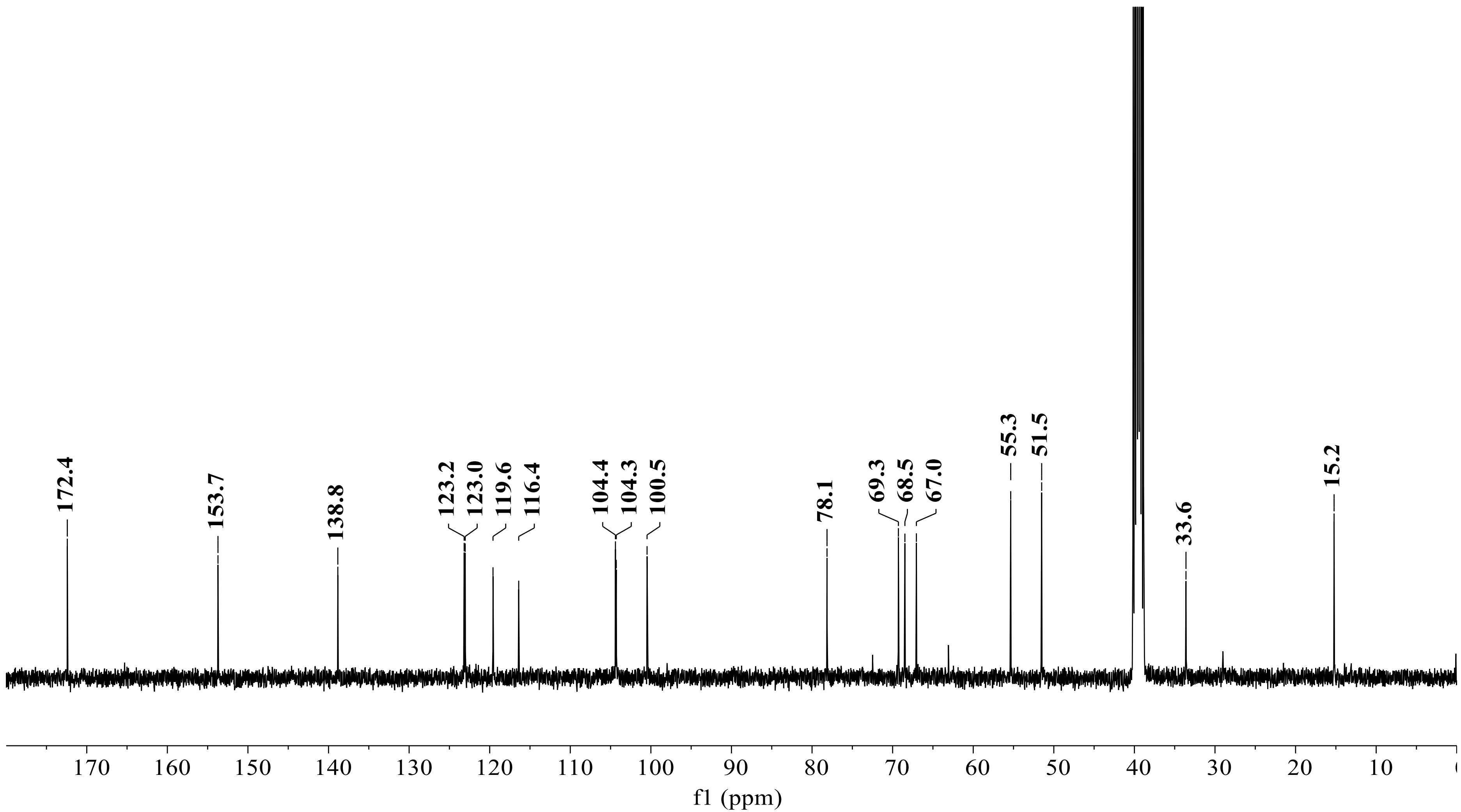

Figure S5: ^{13}C NMR spectrum (100 MHz) of **1** in DMSO-d_6.

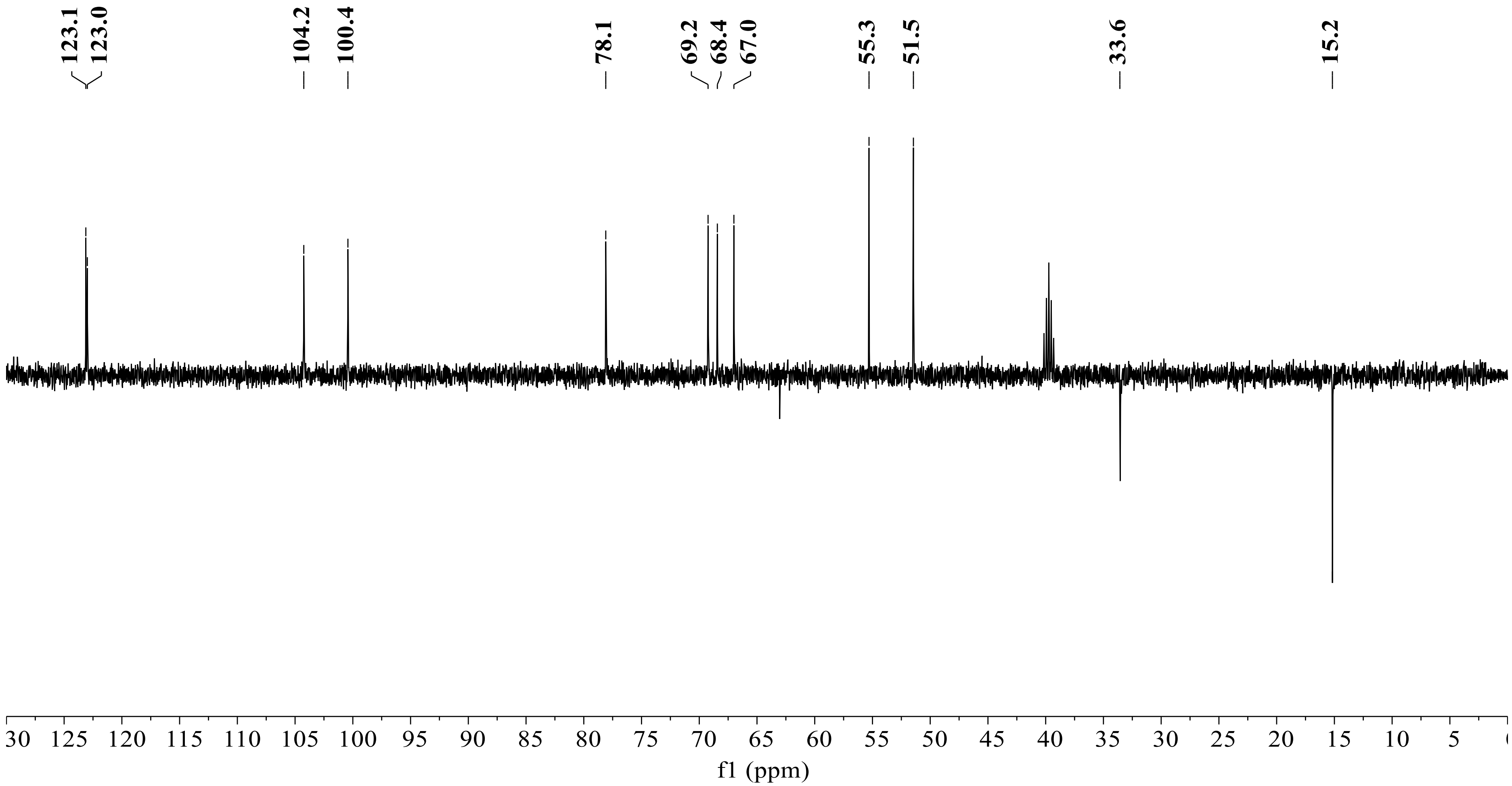

Figure S6: DEPT135 spectrum of **1** in DMSO-d_6.

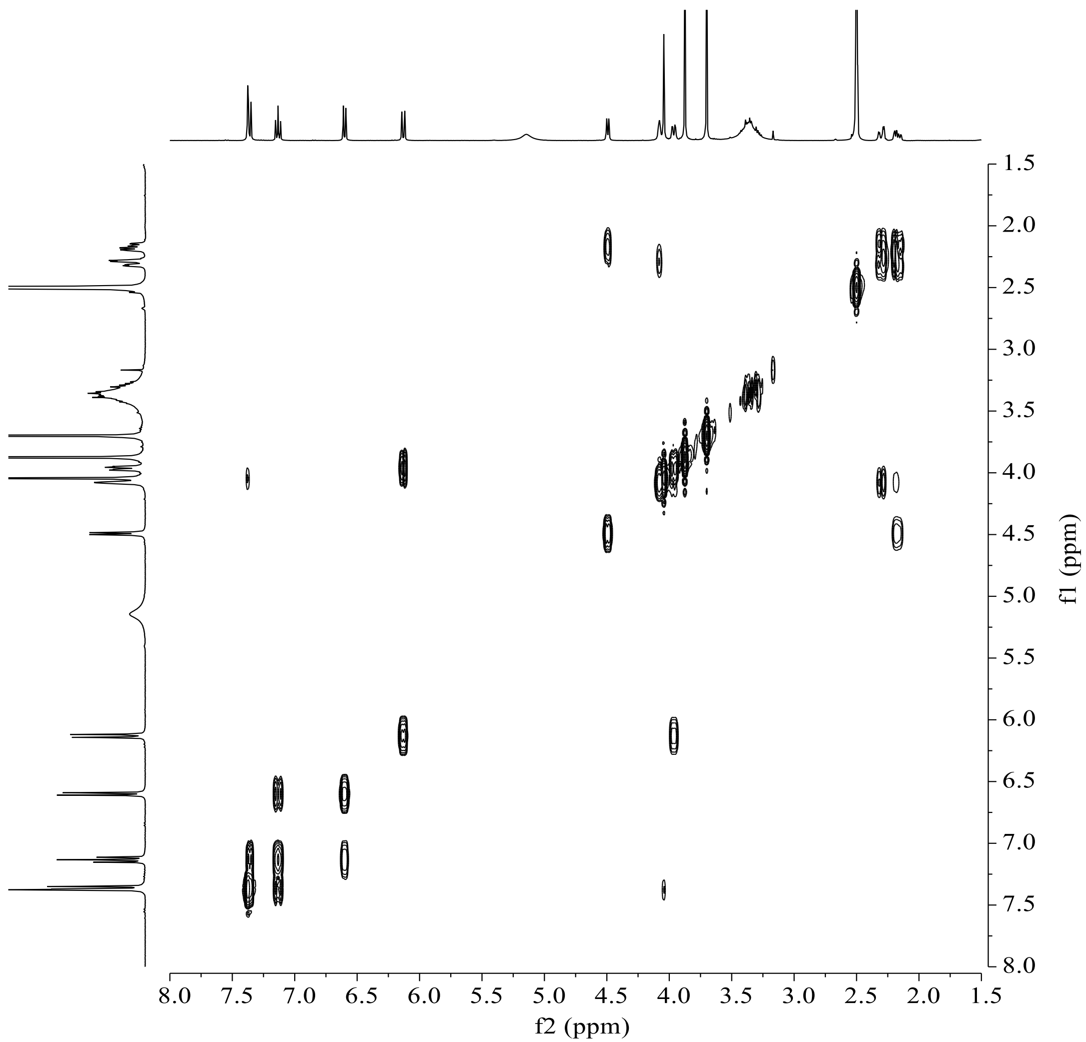

Figure S7: $^{1}H,^{1}H$ COSY spectrum of **1** in DMSO-d_6.

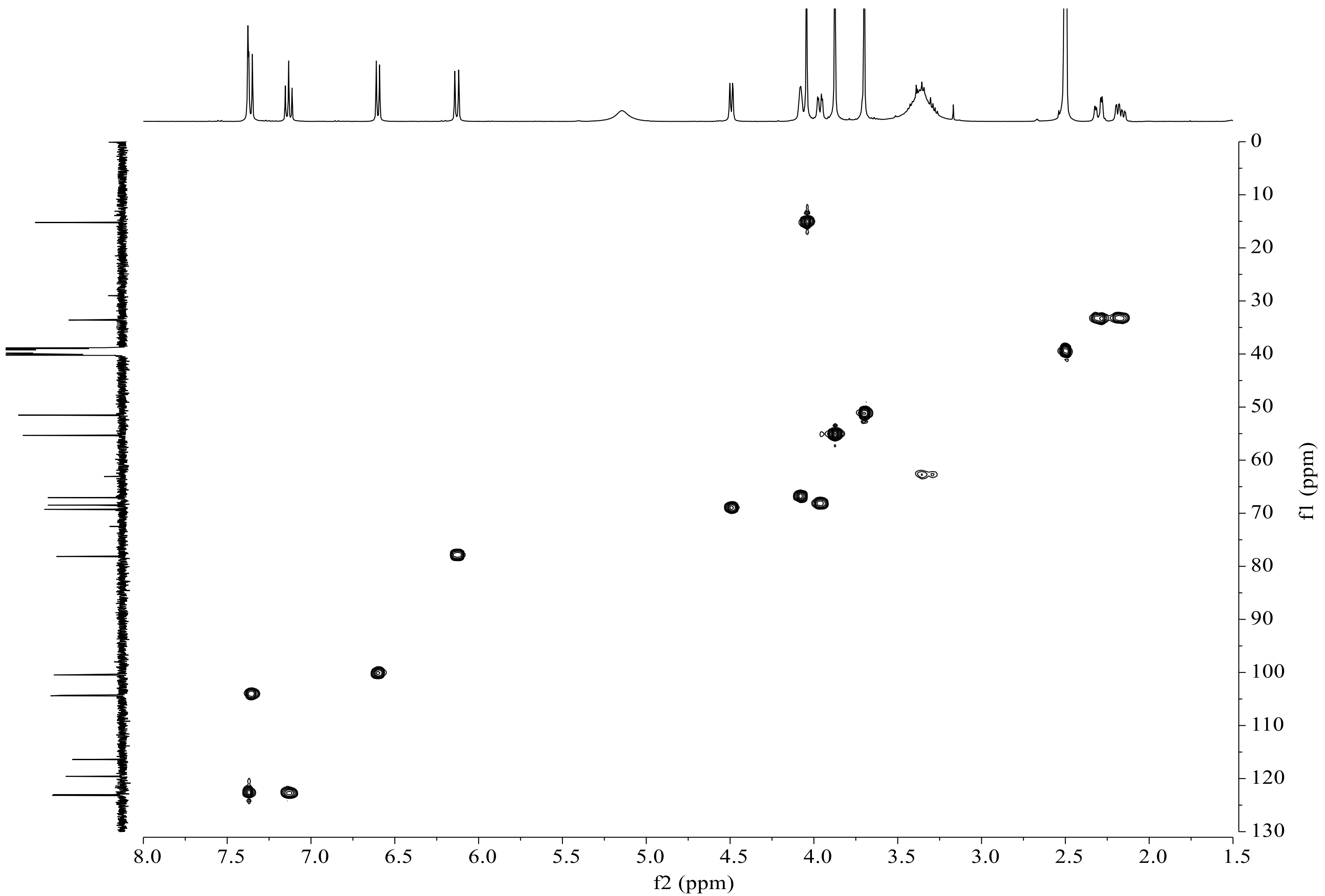

Figure S8: HSQC spectrum of **1** in DMSO-d_6.

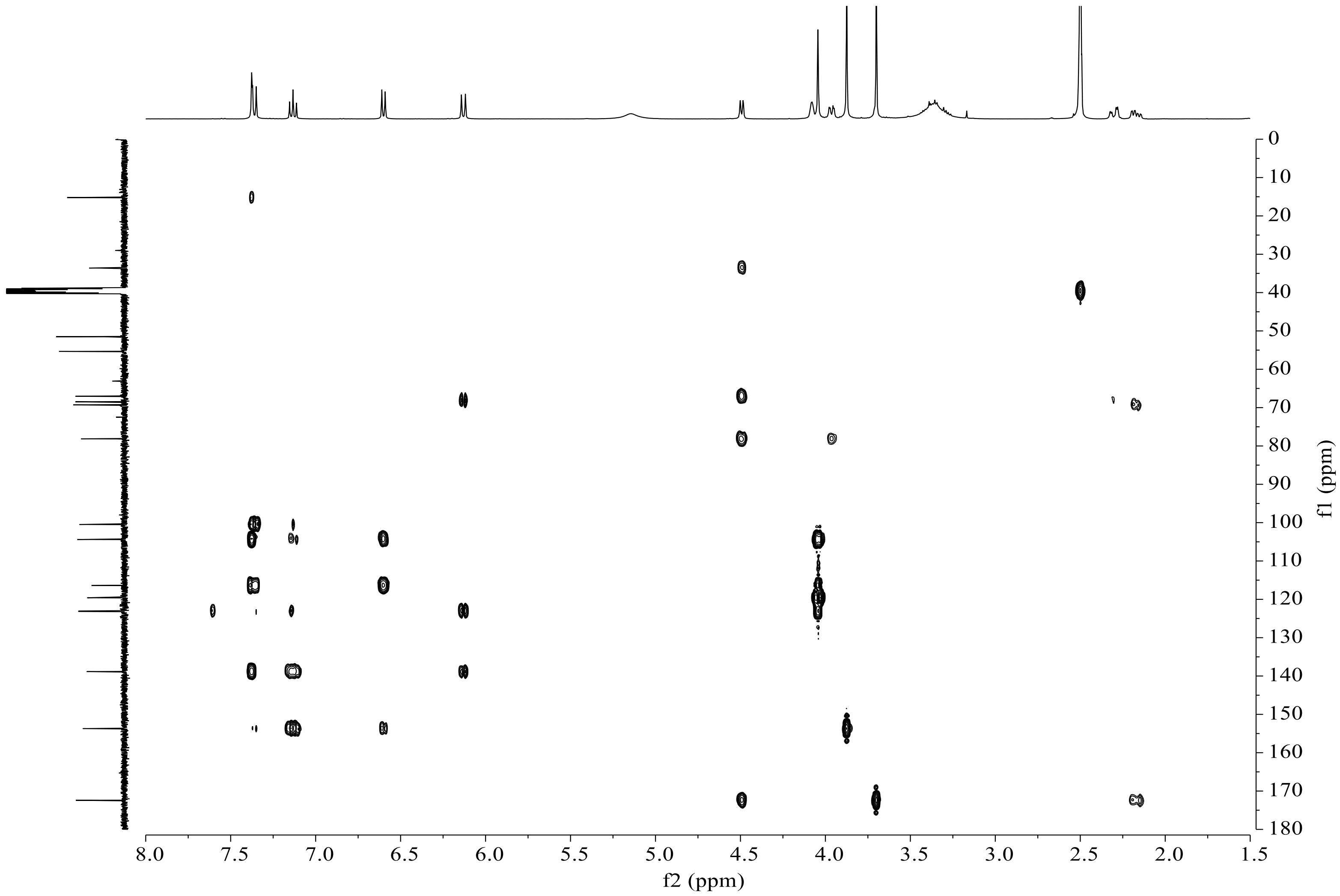

Figure S9: HMBC spectrum of **1** in DMSO-d_6.

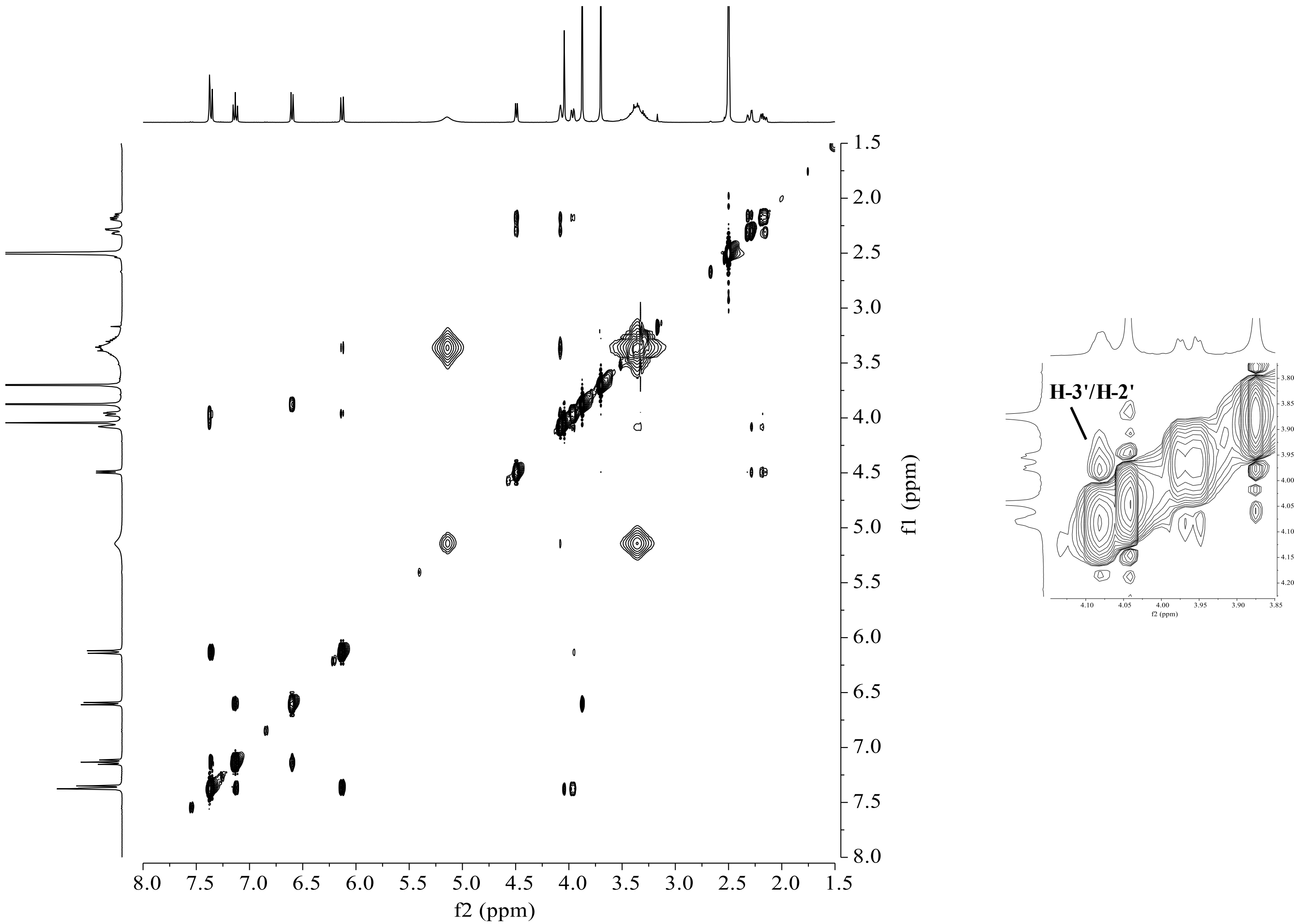

Figure S10: NOESY spectrum of **1** in DMSO-d_6.

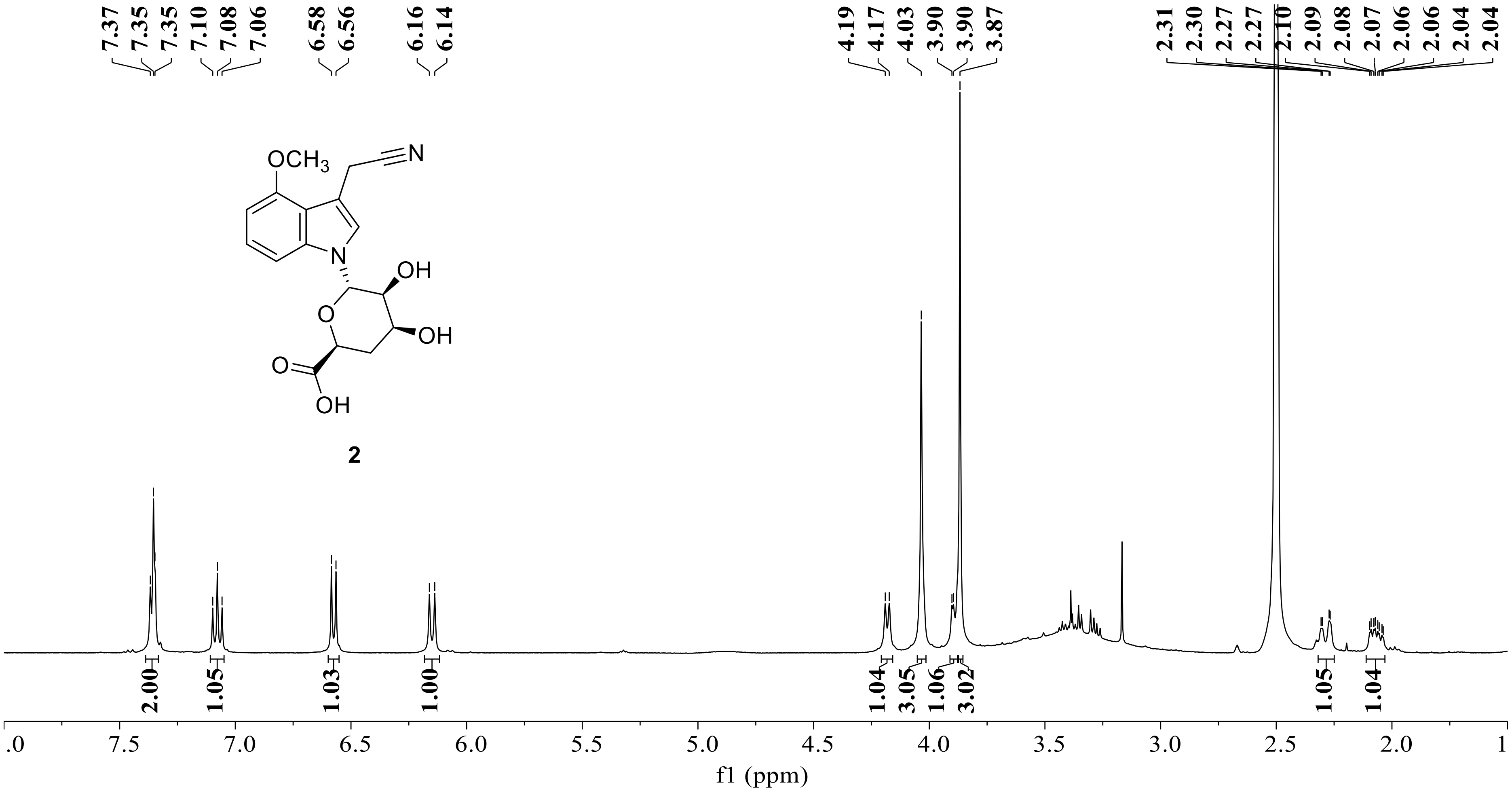

Figure S11: ^{1}H NMR spectrum (400 MHz) of **2** in DMSO-d_6.

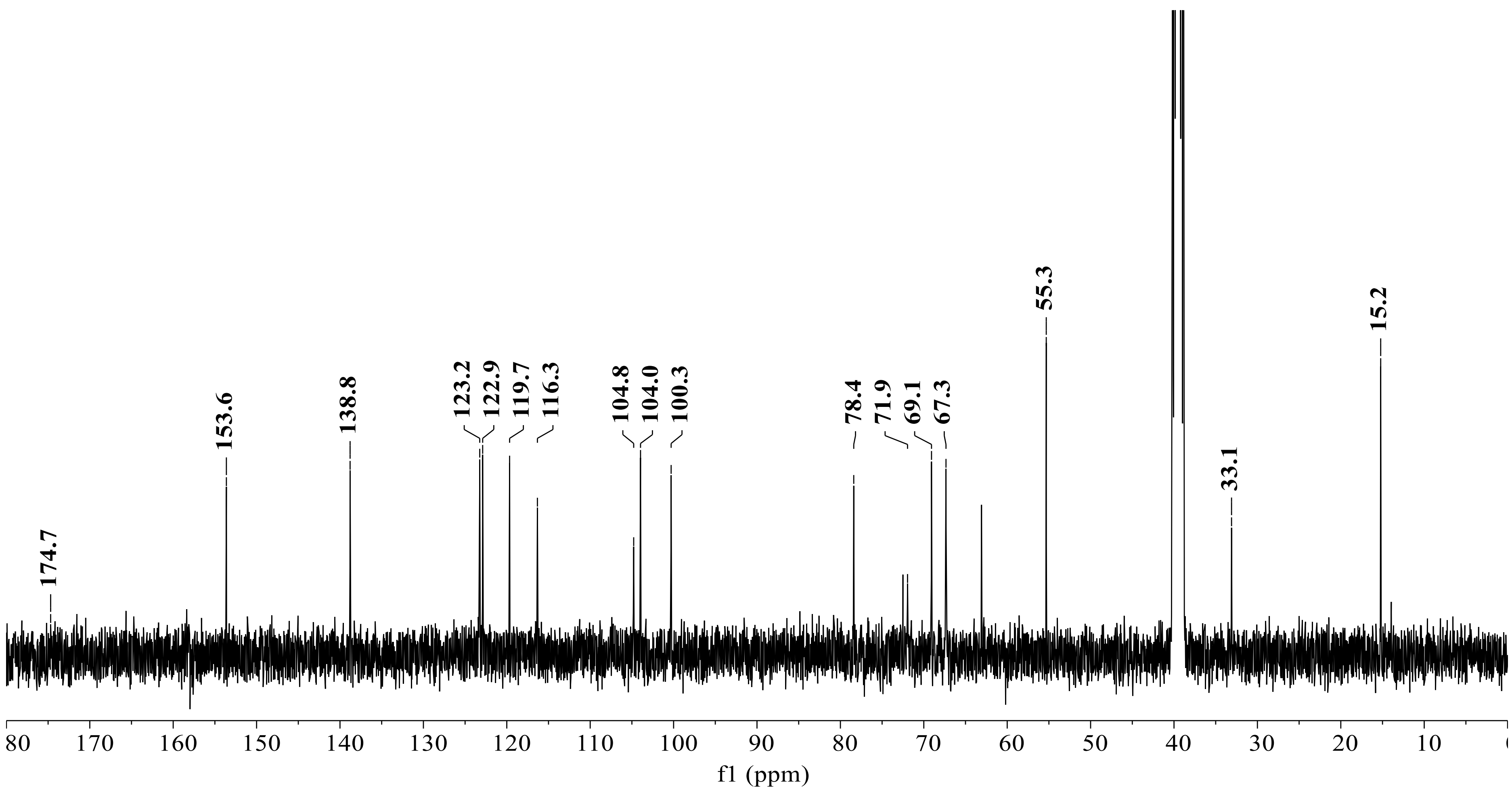

Figure S12: ^{13}C NMR spectrum (100 MHz) of **2** in DMSO-d_6.

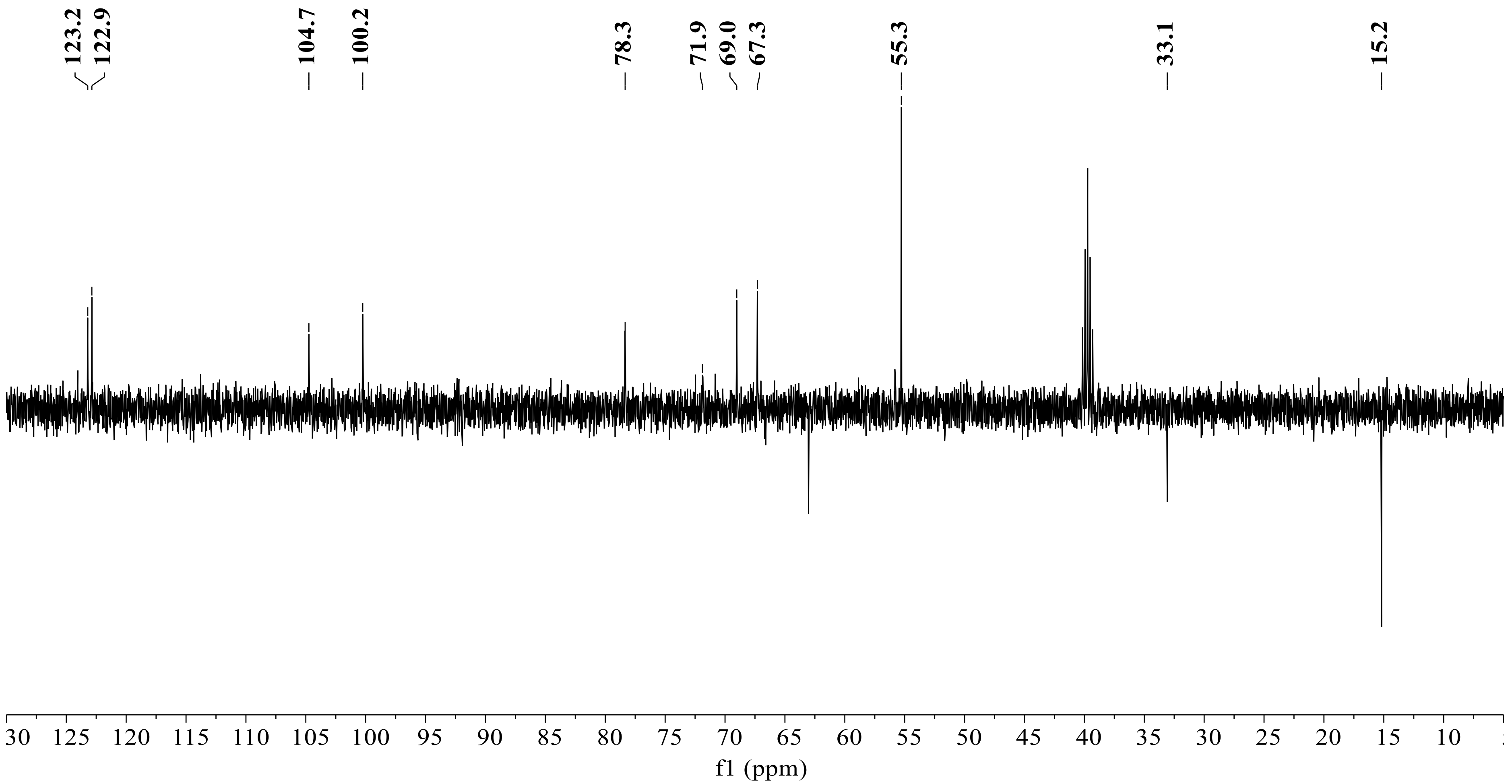

Figure S13: DEPT135 spectrum of **2** in DMSO-d_6.

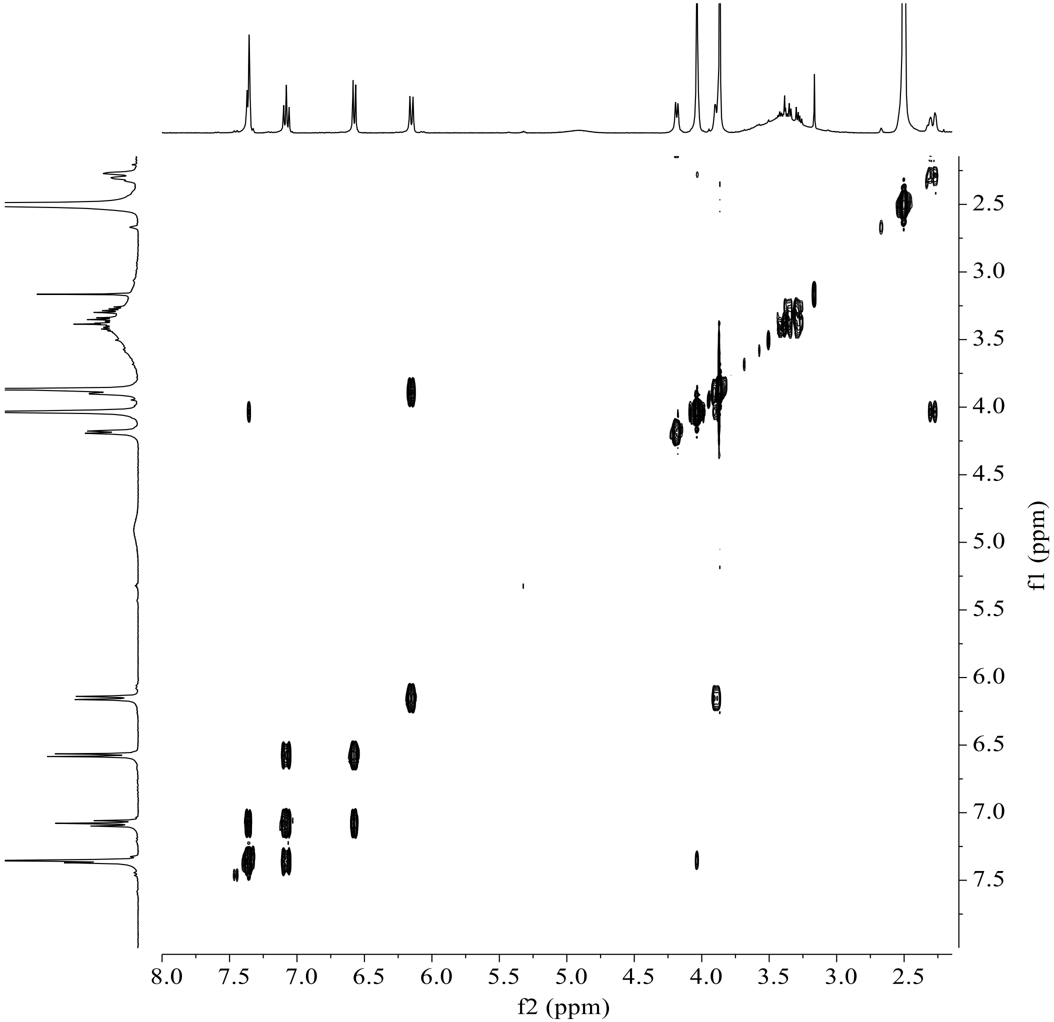

Figure S14: $^{1}H,^{1}H$ COSY spectrum of **2** in DMSO-d_6.

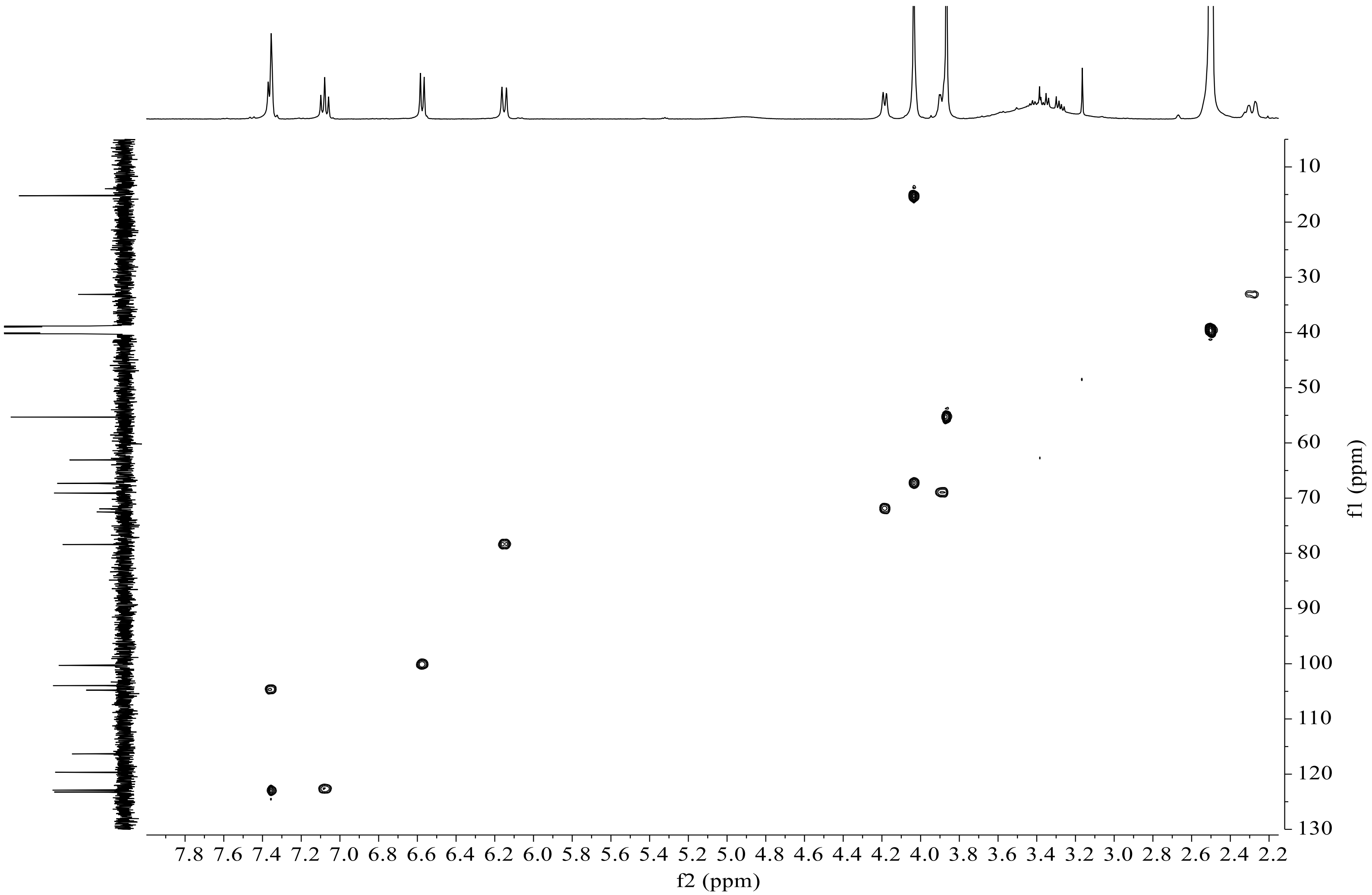

Figure S15: HSQC spectrum of **2** in DMSO-d_6.

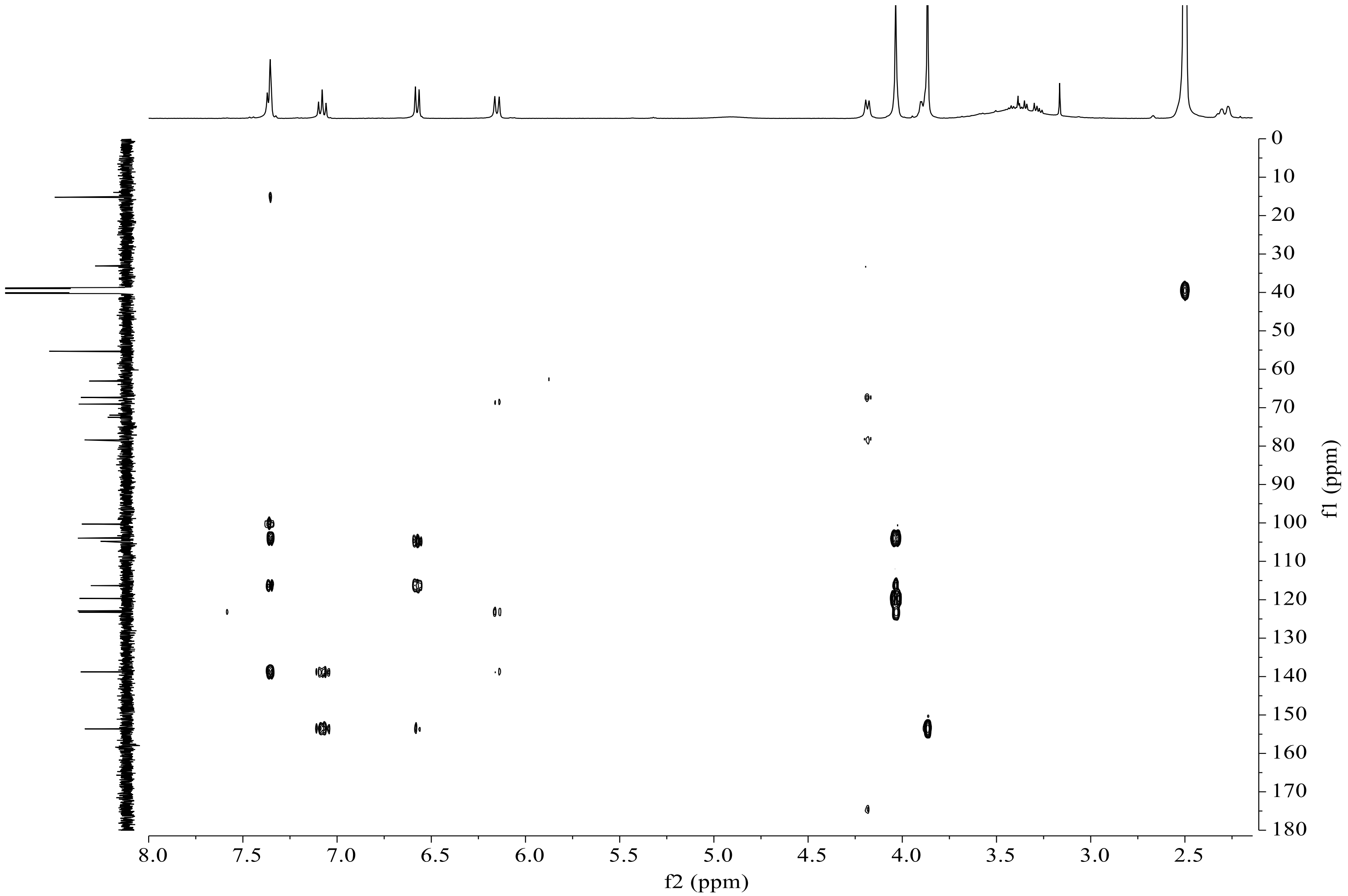

Figure S16: HMBC spectrum of **2** in DMSO-d_6.

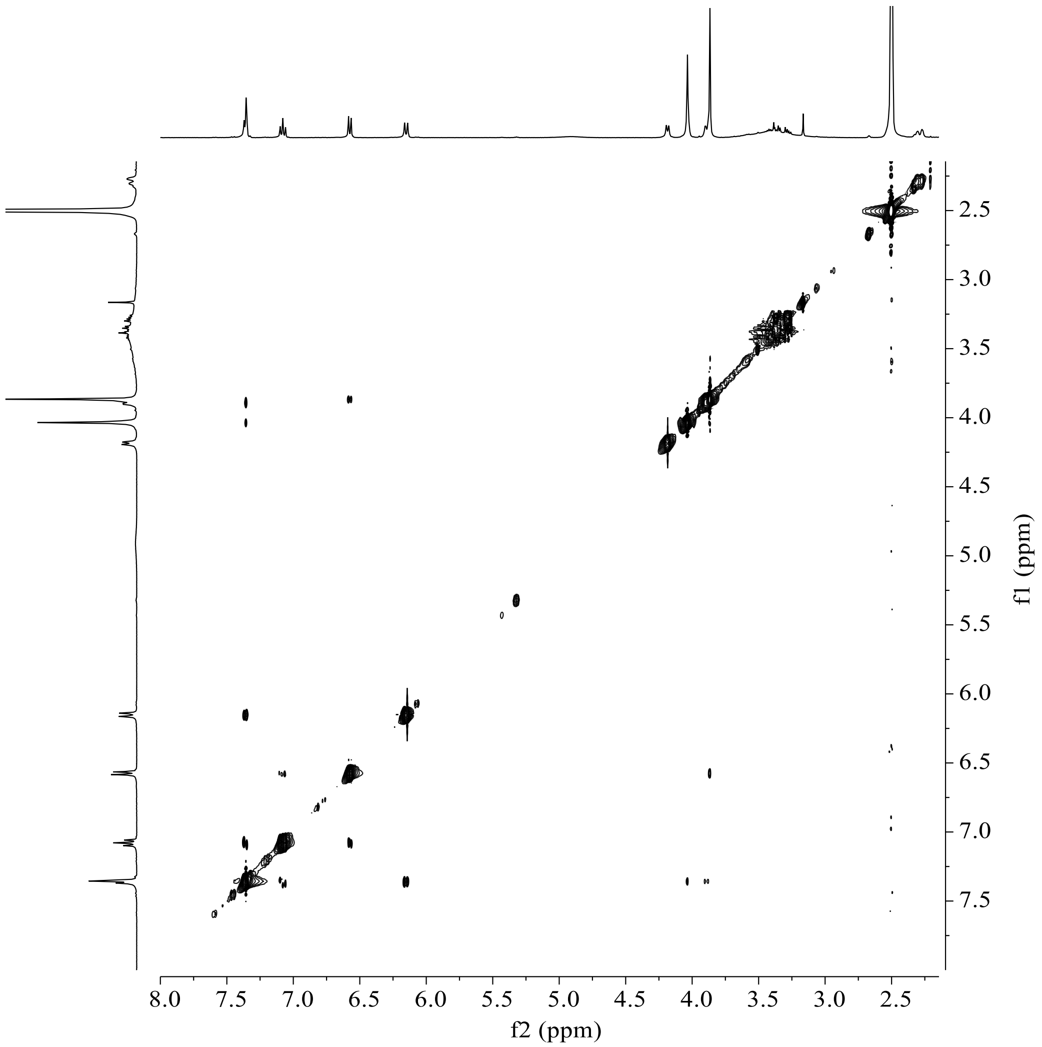

Figure S17: NOESY spectrum of **2** in DMSO-d_6.

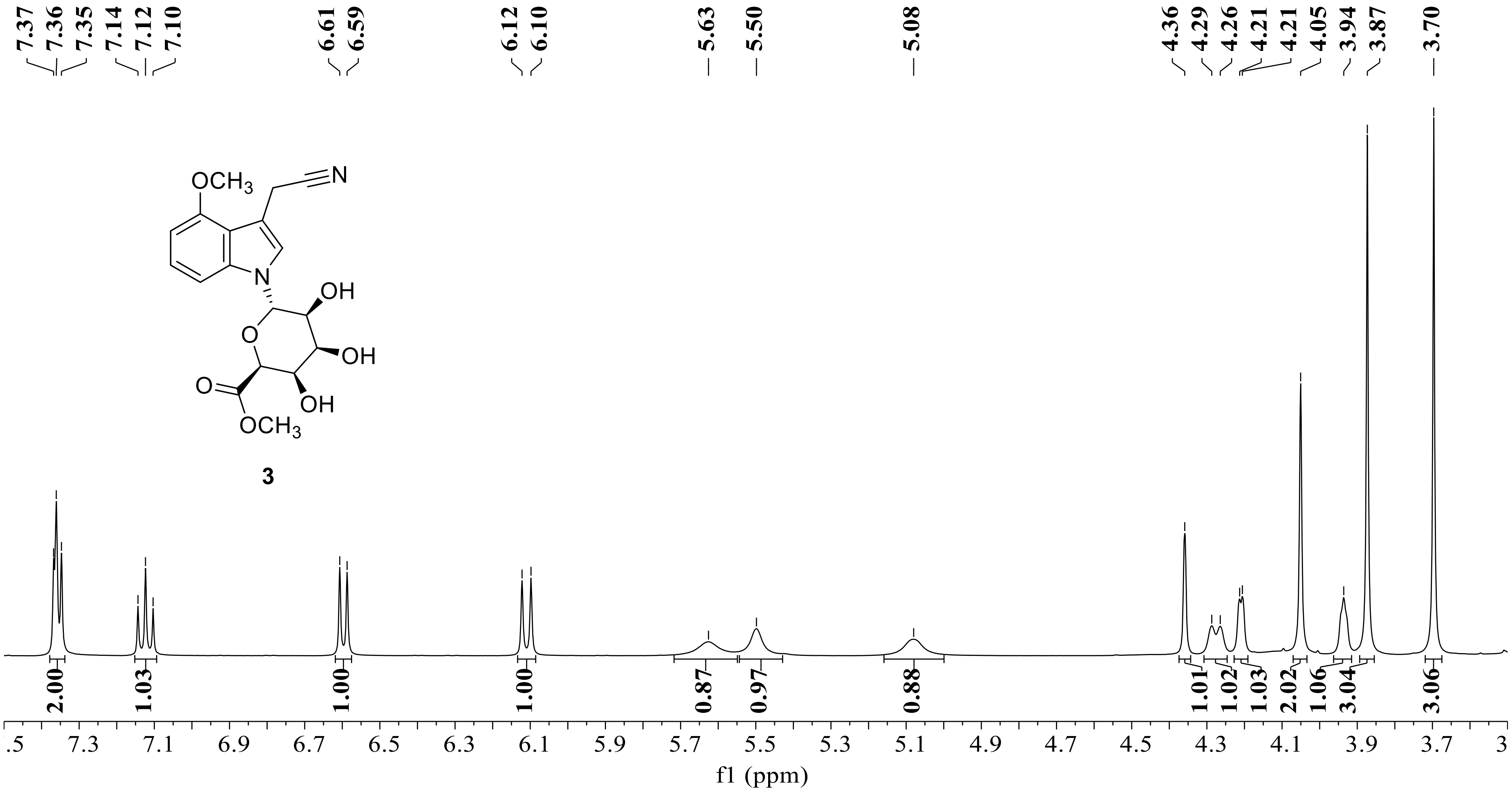

Figure S18: ^{1}H NMR spectrum (400 MHz) of **3** in DMSO-d_6.

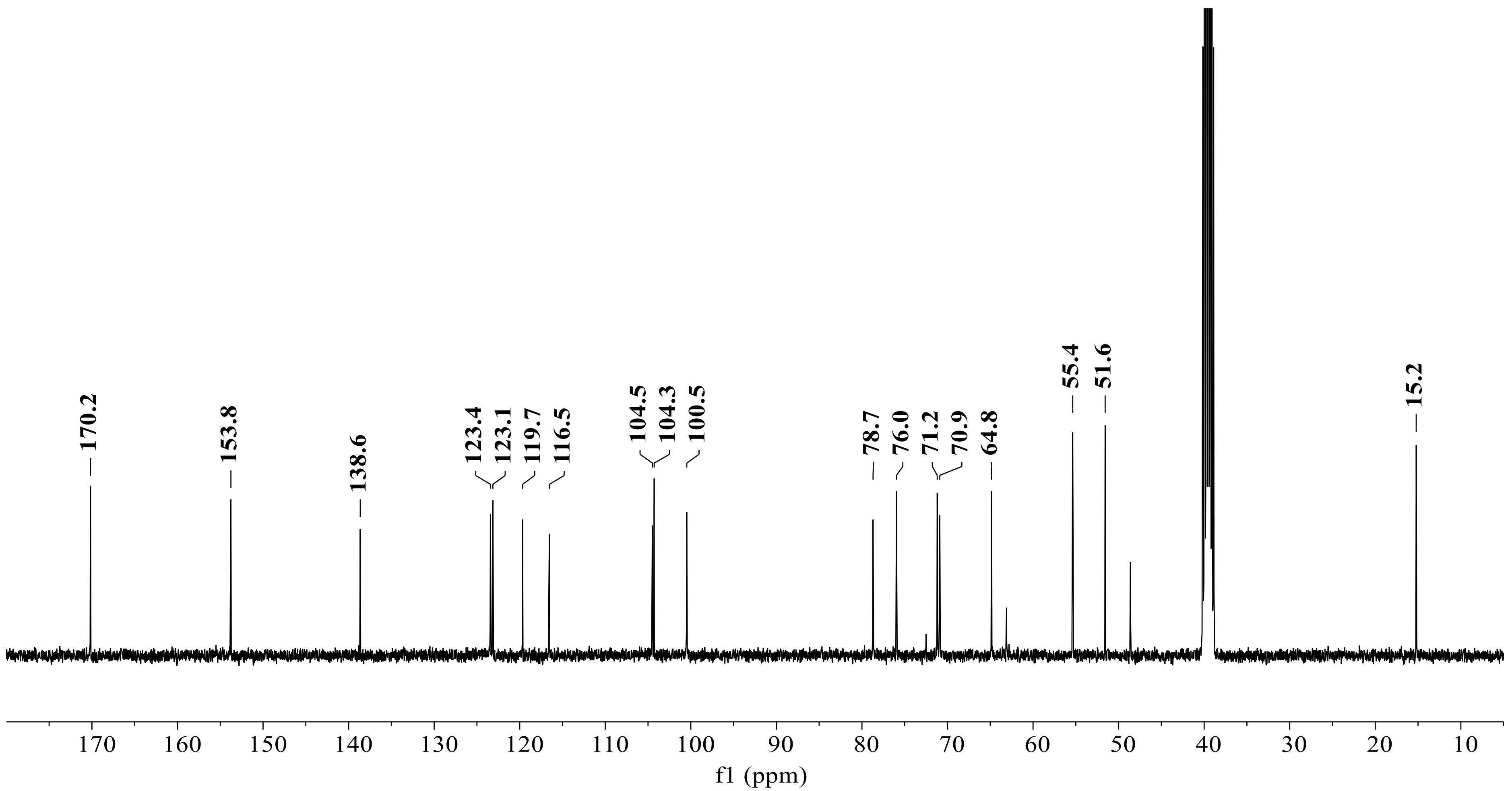

Figure S19: ^{13}C NMR spectrum (100 MHz) of **3** in DMSO-d_6.

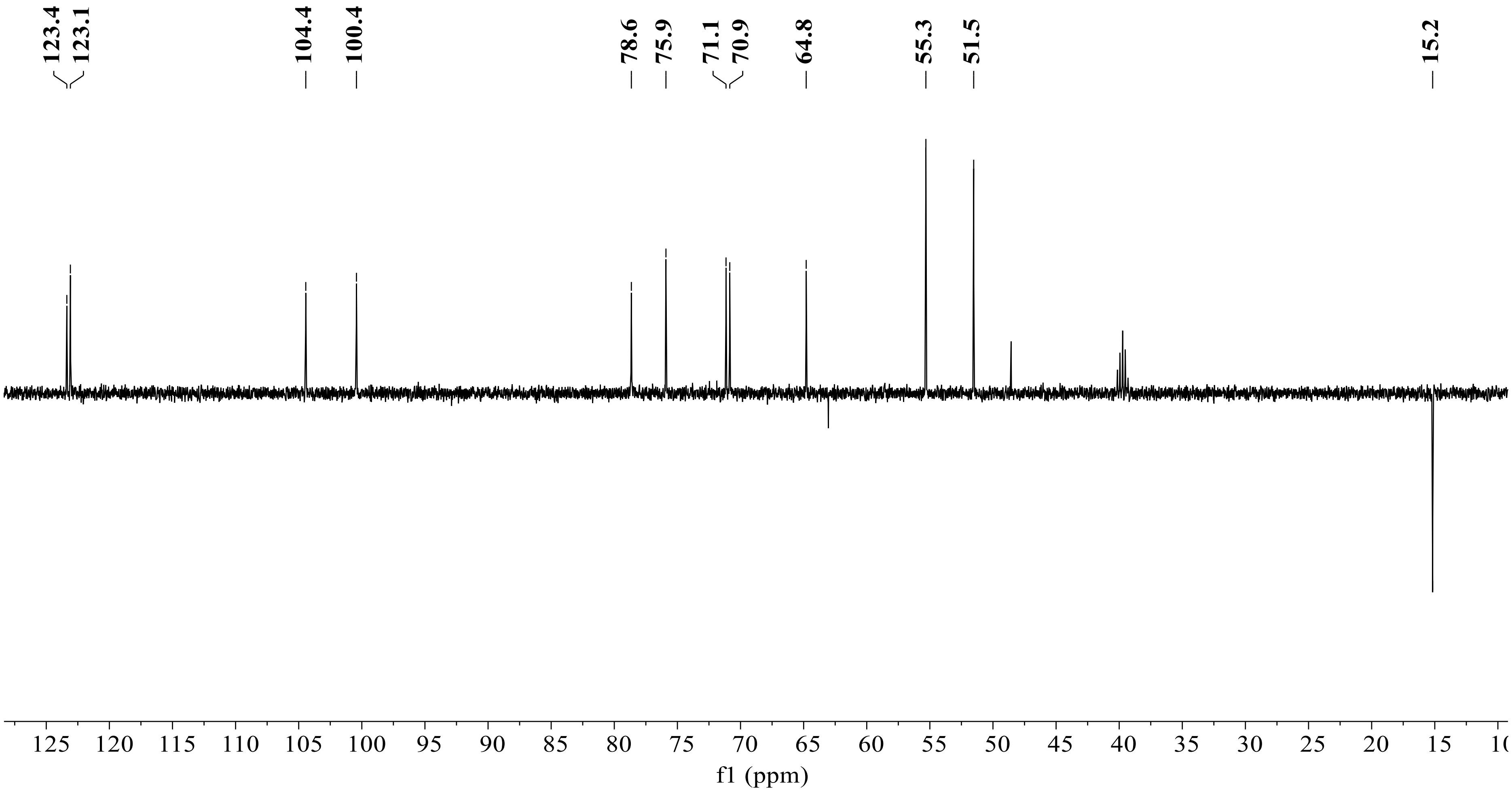

Figure S20: DEPT135 spectrum of **3** in DMSO-d_6.

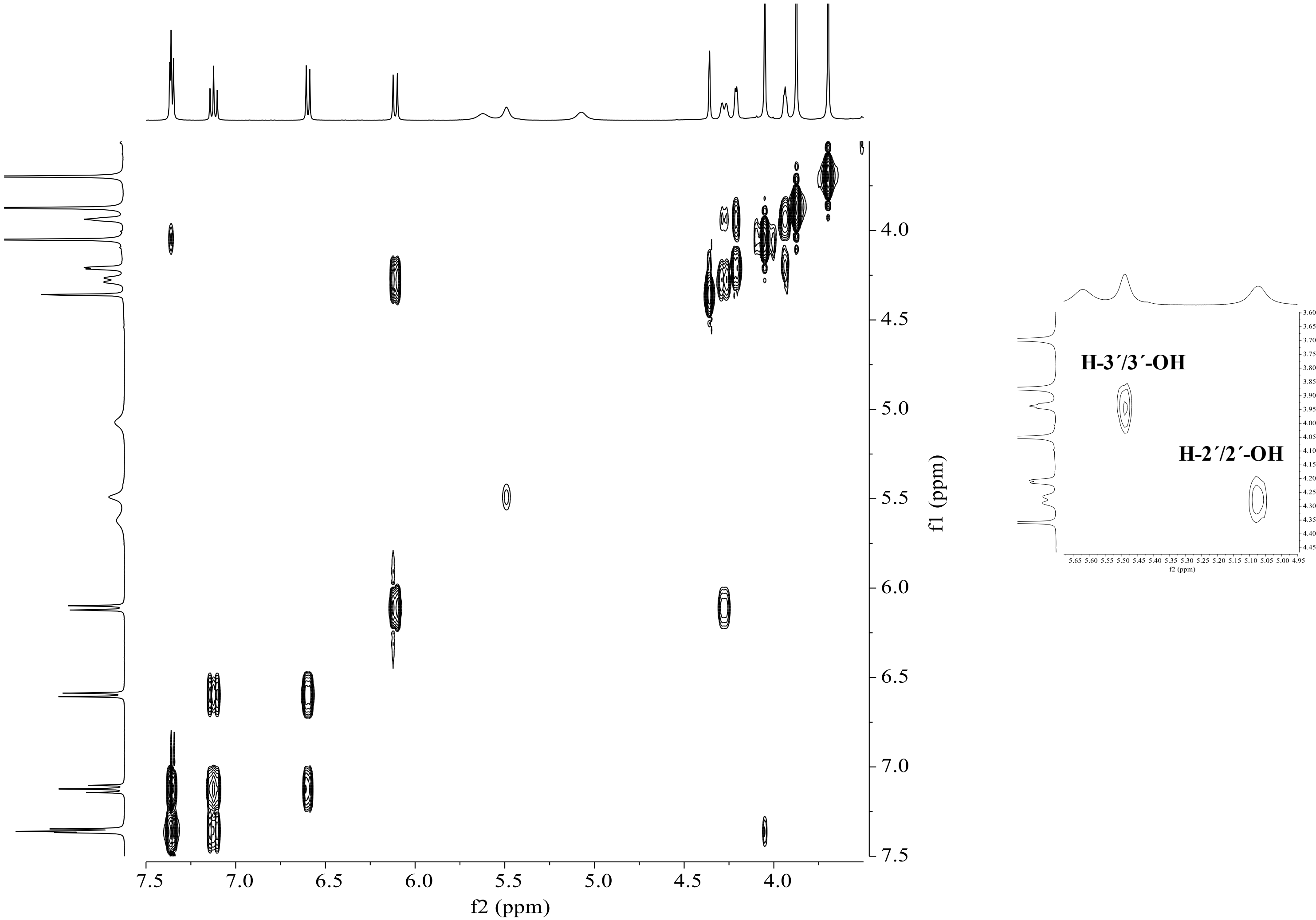

Figure S21: $^1H,^1H$ COSY spectrum of **3** in DMSO-d_6.

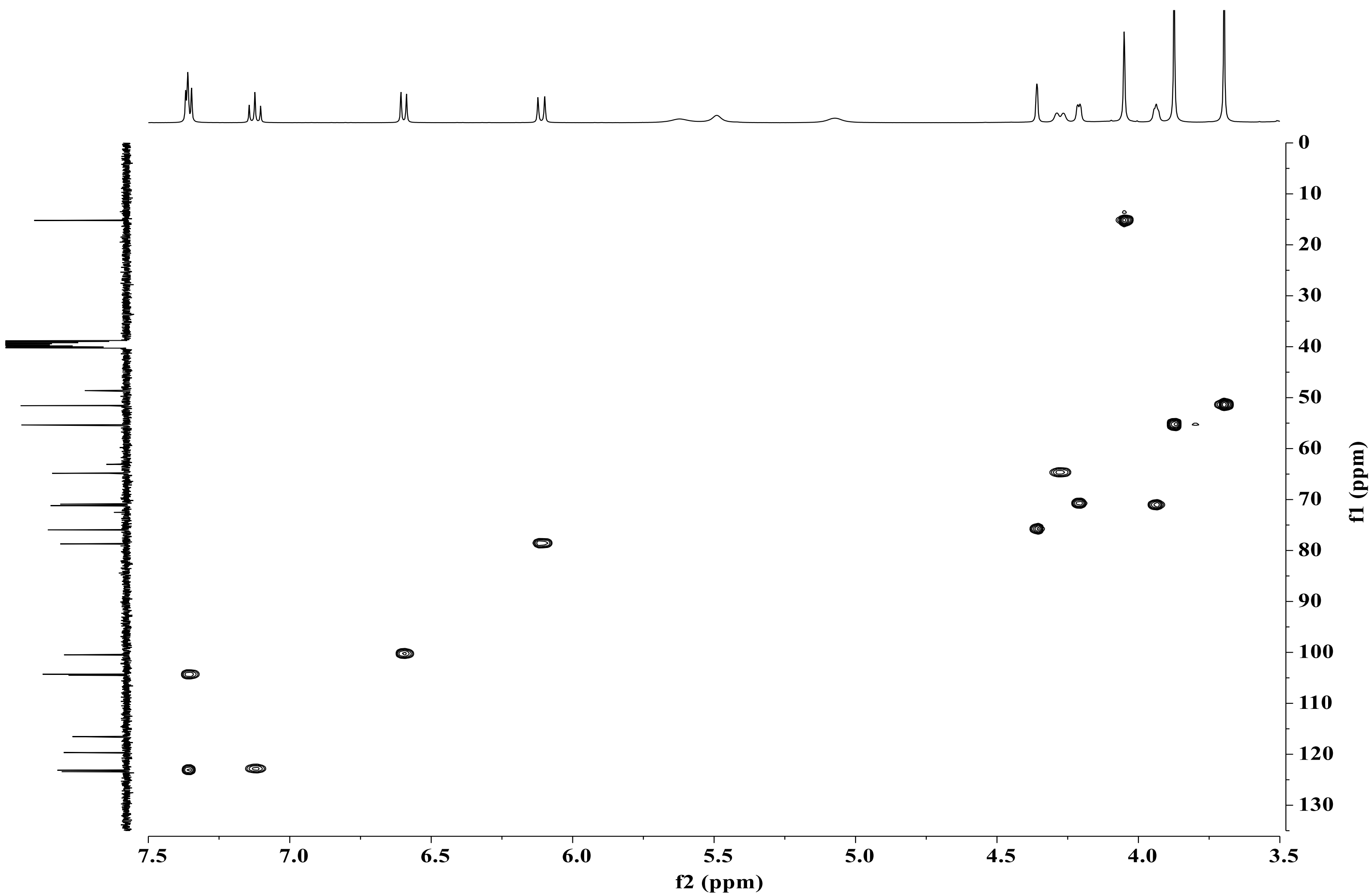

Figure S22: HSQC spectrum of **3** in DMSO-d_6.

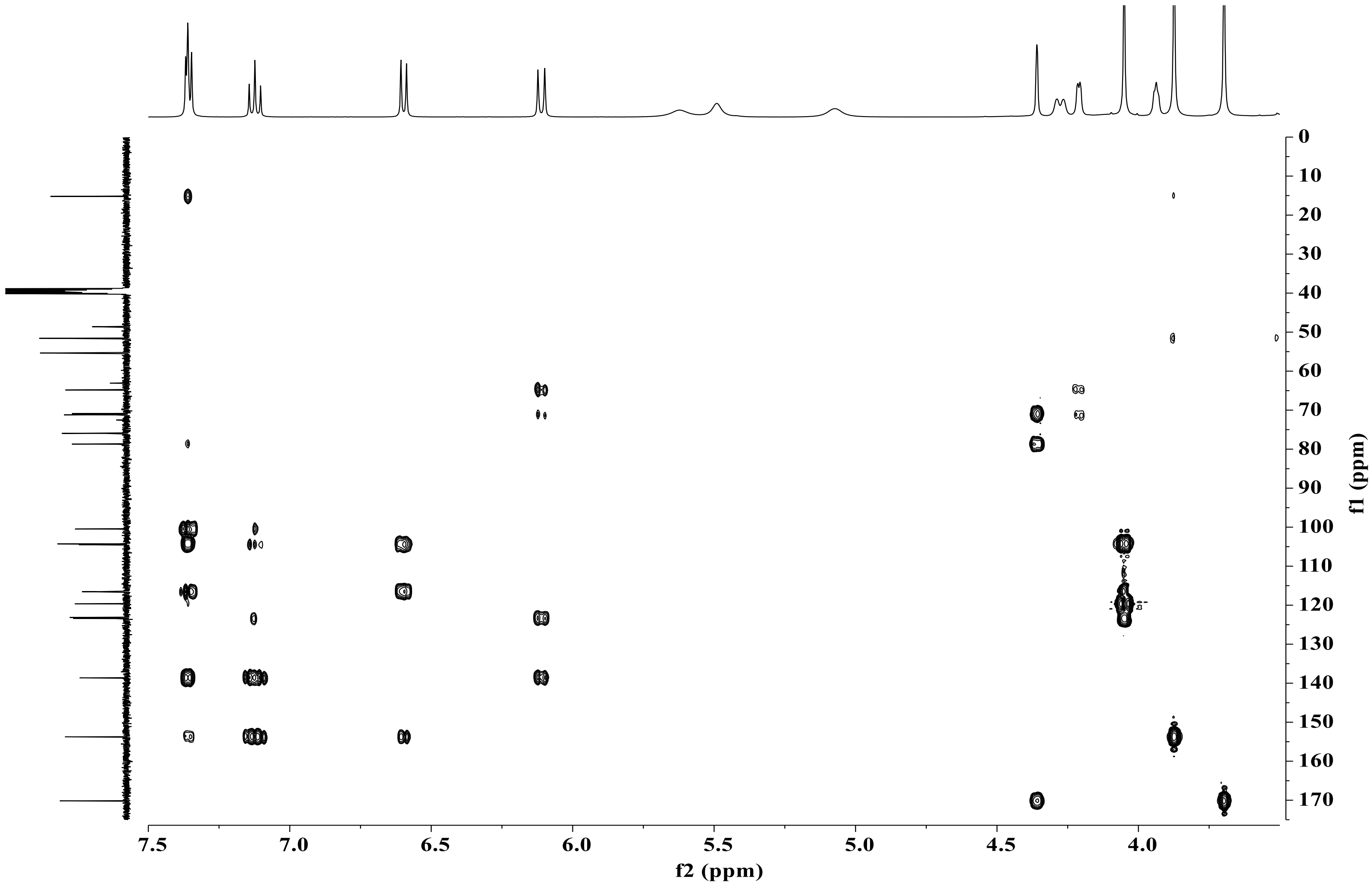

Figure S23: HMBC spectrum of **3** in DMSO-d_6.

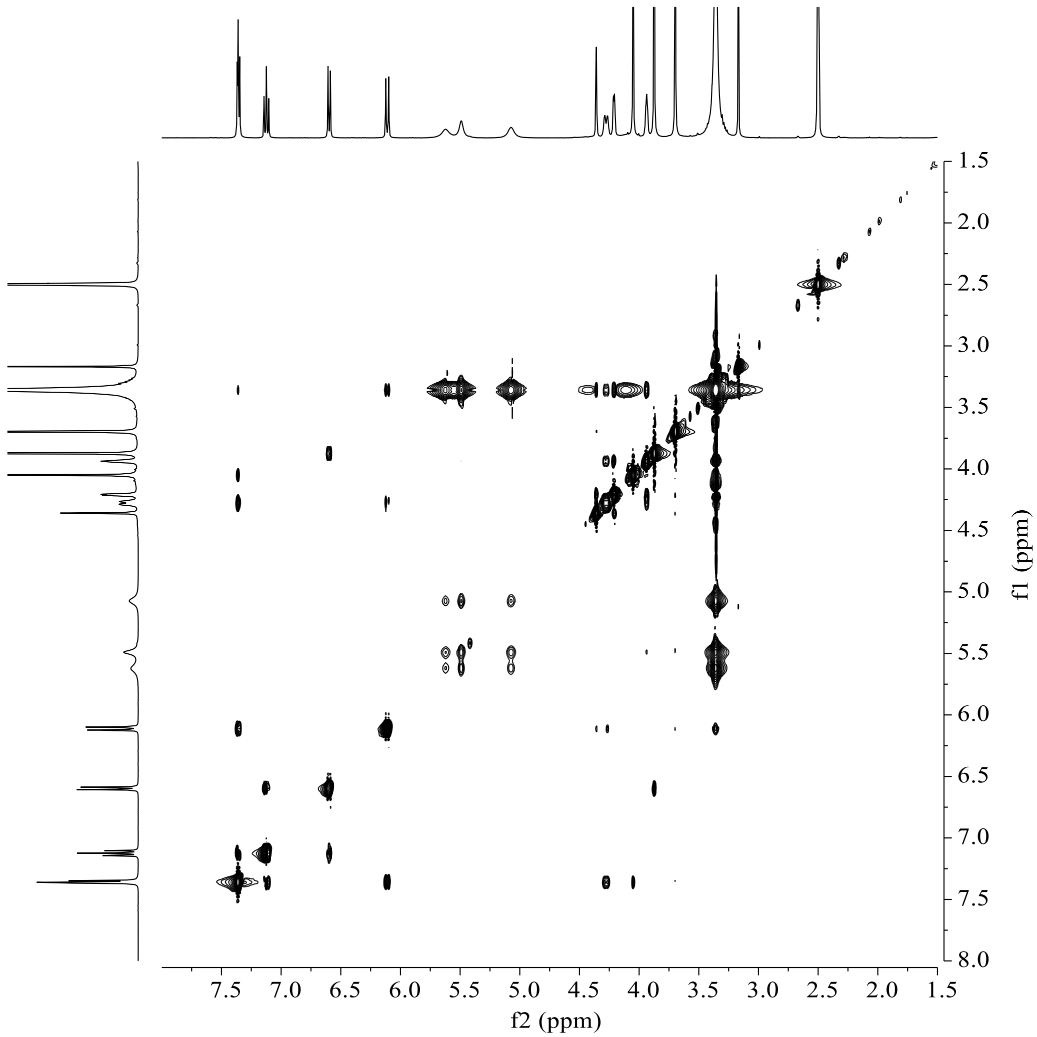

Figure S24: NOESY spectrum of **3** in DMSO-d_6.
